# Complete mapping of viral escape from neutralizing antibodies

**DOI:** 10.1371/journal.ppat.1006271

**Published:** 2017-03-13

**Authors:** Michael B. Doud, Scott E. Hensley, Jesse D. Bloom

**Affiliations:** 1 Basic Sciences and Computational Biology Program, Fred Hutchinson Cancer Research Center, Seattle, Washington, United States of America; 2 Department of Genome Sciences, University of Washington, Seattle, Washington, United States of America; 3 Medical Scientist Training Program, University of Washington, Seattle, Washington, United States of America; 4 Department of Microbiology, Perelman School of Medicine, University of Pennsylvania, Philadelphia, Pennsylvania, United States of America; University of Michigan, UNITED STATES

## Abstract

Identifying viral mutations that confer escape from antibodies is crucial for understanding the interplay between immunity and viral evolution. We describe a high-throughput approach to quantify the selection that monoclonal antibodies exert on all single amino-acid mutations to a viral protein. This approach, mutational antigenic profiling, involves creating all replication-competent protein variants of a virus, selecting with antibody, and using deep sequencing to identify enriched mutations. We use mutational antigenic profiling to comprehensively identify mutations that enable influenza virus to escape four monoclonal antibodies targeting hemagglutinin, and validate key findings with neutralization assays. We find remarkable mutation-level idiosyncrasy in antibody escape: for instance, at a single residue targeted by two antibodies, some mutations escape both antibodies while other mutations escape only one or the other. Because mutational antigenic profiling rapidly maps all mutations selected by an antibody, it is useful for elucidating immune specificities and interpreting the antigenic consequences of viral genetic variation.

## Author summary

Many viruses evolve rapidly, and this evolution sometimes enables them to escape antibodies that would otherwise neutralize their infectivity. An important aspect of studying this evolution is determining which viral mutations can mediate antibody escape. The classic way of identifying such mutations is to select or test them one by one. However, a vast number of possible mutations can be made to a virus. For instance, there are over 10,000 single amino-acid mutations that can be made to the most abundant surface protein of influenza virus, hemagglutinin. This is too many to test one by one, and so all previous studies of antibody escape have examined just a fraction of the possible amino-acid mutations to any given viral protein. Here we describe a new approach to quantify the selection that an antibody exerts on *all* these mutations in a single experiment. This approach enables us to reproducibly and sensitively identify mutations that affect antibody neutralization—for instance, at individual sites in hemagglutinin, we can distinguish which of several different mutations have the largest effect on antibody escape. The ability to completely map viral escape from antibodies opens the door to much more detailed characterization of viral antigenic evolution.

## Introduction

Host immunity drives the evolution of many viruses. For instance, potent immunity against influenza virus is provided by antibodies against hemagglutinin (HA), the virus’s most abundant surface protein [1]. Unfortunately, these antibodies also select amino-acid substitutions in the HA of human seasonal influenza A virus at a rate of over two per year [2, 3]. This rapid evolution degrades the effectiveness of anti-influenza immunity, and is a major reason why humans are repeatedly re-infected over their lifetimes. Extensive antigenic variation is also a hallmark of several other medically relevant viruses, most prominently HIV. Efforts to induce immunity to such viruses must therefore account for antigenic variation, either by targeting vaccines against current circulating viral strains [4, 5] or developing methods to administer [6, 7] or elicit [8, 9] antibodies that recognize a broad range of strains. An important component of these efforts is identifying which viral mutations escape neutralization by specific antibodies.

The classic approach for identifying such mutations is to select individual viral mutants that are resistant to neutralization by antibodies. For instance, escape-mutant selections with a panel of monoclonal antibodies were used to broadly define major antigenic regions of influenza HA [10–12]. However, each such selection typically only identifies one of potentially many mutations that escape an antibody, with a strong bias towards whichever mutations happen to be prevalent in the initial viral stock. Therefore, escape-mutant selections provide an incomplete picture of the ways that a virus can escape an antibody.

Another approach is to individually test antibody binding or neutralization for each member of a panel of viral variants. However, there are ∼10^4^ single amino-acid mutants to a 500-residue viral protein, so individually creating and testing all of them is a daunting task. Therefore, even the most ambitious such studies limit themselves to a small fraction of the possible point mutations, such as by only testing mutations to alanine [13–15]. But as the current work will underscore, the antigenic effect of mutating a residue to one amino acid can be poorly predictive of the effects of mutating the same residue to another amino acid. Furthermore, the difficulty in individually generating and testing large numbers of viral variants means that such studies often use simpler assays (e.g., hemagglutination-inhibition, pseudovirus neutralization, or protein binding) that can be imperfect surrogates for how well a mutation enables a replication-competent virus to escape antibody neutralization [16–18].

A complete structural definition of the interface between an antibody and antigen can be obtained using methods such as X-ray crystallography. However, obtaining such structures remains non-trivial, particularly since viral surface proteins are often heavily glycosylated [19] and sometimes conformationally heterogeneous [20]. In addition, structural definitions do not reveal which mutations actually escape antibody neutralization. Mutations at only a subset of the residues in the antibody-antigen interface actually disrupt binding [21–24], a “hot spot” phenomenon observed in protein-protein interfaces more generally [25–27].

Here we use massively parallel experiments to rapidly map all single amino-acid mutations to HA that enable influenza virus to escape from four neutralizing antibodies. Our approach involves imposing antibody selection on virus libraries generated from all amino-acid point mutants of HA, and using deep sequencing to quantify the selection on every mutation in the context of actual replication-competent virus. The resulting comprehensive map of antibody escape reveals remarkable mutation-level idiosyncrasy for each antibody: for instance, at many residues only some of the possible amino-acid mutations confer escape, and two antibodies targeting the same residue elicit unique profiles of escape mutations. Mutational antigenic profiling therefore enables complete and high-resolution mapping of viral antibody escape mutations.

## Results

### Reproducible measurement of antibody selection on all amino-acid point mutations to influenza HA

**Fig 1:**
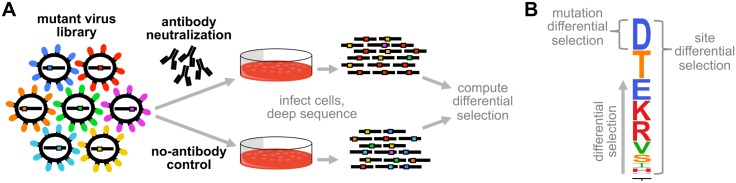
Mutational antigenic profiling. **(A)** Libraries of viruses carrying all amino-acid point mutants of a protein that support viral replication are incubated with or without antibody, and used to infect cells. Viral RNA is extracted from cells and accurately deep sequenced to quantify the frequency of each mutation in the antibody-selected and no-antibody control samples. **(B)** Differential selection is defined as the logarithm of the enrichment of each mutation in the antibody-selected sample versus the control. In the logo plots, the height of each letter is proportional to the differential selection for that amino-acid. The site differential selection is the total height of the logo stack at that site (the sum of mutation differential selection values). Only positive differential selection (corresponding to mutations enriched by selection) is shown. Logo plot letters are colored by physicochemical properties of amino-acids.

To quantify the selection that neutralizing antibodies exert on all single amino-acid mutations to a viral protein, we developed the mutational antigenic profiling strategy shown in Fig 1. A library of viruses is generated that contains all amino-acid point mutants of the protein that are compatible with viral replication. This library is incubated with or without a neutralizing antibody, and then used to infect cells. Deep sequencing of cellular RNA measures the frequency of each mutation among the viral variants that infect cells in the presence or absence of antibody, with molecular barcoding used to increase the sequencing accuracy. We quantify the *differential selection* for each mutation as the logarithm of its enrichment in the antibody-treated virus library relative to the no-antibody control, and display these data as in Fig 1B. In the analysis that follows, we only consider mutations with positive differential selection.

We applied mutational antigenic profiling to influenza HA. HA is a 565-residue glycoprotein that forms homo-trimers on the virion surface that are responsible for both receptor-binding and membrane fusion [1]. Current influenza vaccines are designed to induce antibodies against HA, and the strains that compose these vaccines are chosen annually with the goal of matching the antigenicity of their HAs with those in circulating influenza variants [2–5]. We chose to focus on the HA of an H1N1 strain (A/WSN/1933) that was isolated from humans early in the study of influenza and then serially passaged in the lab. Our reason for choosing this strain is that classic escape-mutant selections have extensively characterized the antigenicity of closely related HAs [11, 12], enabling us to compare our results to those obtained using more traditional methods.

The first step in mutational antigenic profiling is creating virus libraries (Fig 1A). A number of techniques have recently been described to create all amino-acid point mutants of a gene in the context of a plasmid [28–30]. The last few years have also seen the description of libraries of replication-competent virus mutants generated by adapting plasmid-based viral reverse-genetics systems to accommodate libraries of mutagenized plasmids [31–35]. We utilized virus libraries created by melding these two techniques to create influenza viruses carrying all HA amino-acid point mutations compatible with viral replication [31, 32].

**Fig 2:**
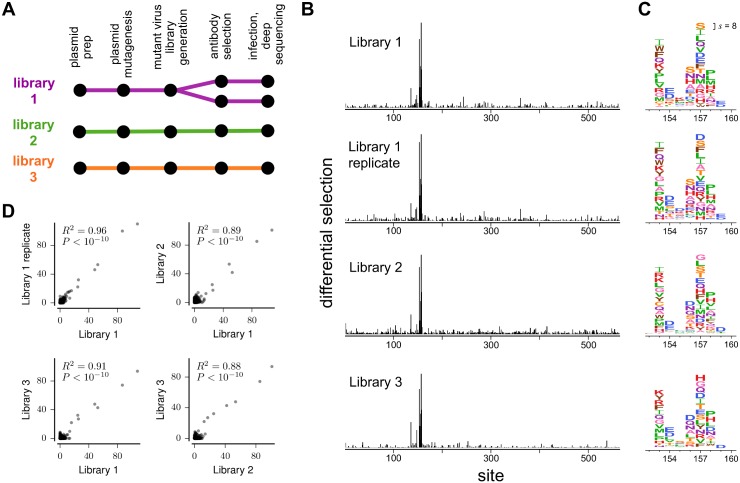
Mutational antigenic profiling with antibody H17-L19 is highly reproducible. **(A)** We performed three biological replicates and one technical replicate. **(B)** Site differential selection across HA is concentrated on the same subset of sites in all replicates. **(C)** Zoomed-in view of selection on the core of the epitope. The height of each letter is proportional to the differential selection for that amino-acid. The same scale is used in all panels of (B) and (C). The scale bar in the upper-right of (C) shows the letter height for a mutation with differential selection of 8, corresponding to 2^8^ = 256-fold enrichment by antibody selection. Residues are numbered sequentially beginning with the initiating methionine; conversions to other numbering schemes are in S1 File. **(D)** Positive site differential selection across all sites is highly correlated among replicates. Each point represents positive site differential selection at one site; correlation coefficients are Pearson’s R. Data is shown for selections with antibody H17-L19 at 10 *μ*g/ml.

We initially selected these libraries with a monoclonal antibody (H17-L19) targeting the Ca2 antigenic region of HA [11]. We performed three biological replicates using independently generated virus libraries, as well as a technical replicate with one of the libraries (Fig 2A). The rationale for performing biological and technical replicates was to evaluate noise arising both from variability in the virus libraries and stochasticity in the antibody selections.

In each replicate, the antibody exerted strong selection for mutations at a handful of sites, and little selection on the rest of HA (Fig 2B). Fig 2C shows the selection for individual amino-acid mutations in a short region in HA containing most of the epitope. Visual inspection reveals consistent selection across technical and biological replicates. Statistical analysis confirms that the site differential selection is strongly correlated among replicates (Fig 2D).

**Fig 3:**
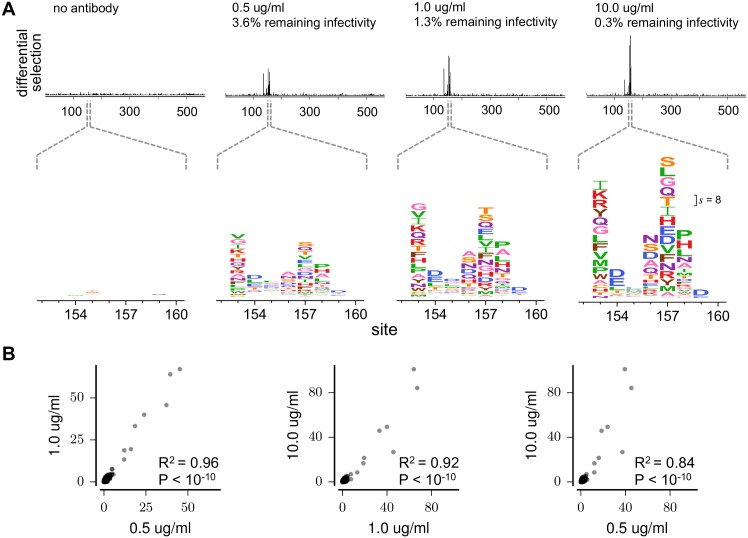
Differential selection by H17-L19 at different antibody concentrations. **(A)** Differential selection increases with antibody concentration. The top plots show site differential selection across HA; the bottom plots show the core of the epitope. All horizontally aligned plots use the same scale. The scale bar in the right-most plot shows the letter height for a mutation with differential selection of 8 (a 256-fold enrichment). The “no antibody” differential selection is computed between two replicate experiments on a single library. **(B)** Site differential selection is correlated between antibody concentrations, although the strength of selection increases at most sites with higher antibody concentration. Each point represents selection at one site in HA; correlation coefficients are Pearson’s R. The data for each concentration is the average across the three biological replicates.

We next asked how the differential selection depended on the concentration of antibody used. Fig 2 shows the results of mutational antigenic profiling at an antibody concentration where the virus libraries retained only 0.3% of their total infectivity. We performed additional experiments using dilutions of antibody that spanned a 20-fold range. Fig 3A shows the selection at each antibody concentration. As expected, there is minimal selection when comparing replicate no-antibody controls. At progressively higher antibody concentrations, differential selection increases at most sites in the epitope, while noise at other sites remains similar to the no-antibody control. However, the increase in differential selection with antibody concentration is not entirely uniform across sites (Fig 3A). Fig 3B shows that despite these complexities, the sites of greatest differential selection are similar across concentrations, indicating that the identification of escape mutations does not strongly depend on antibody concentration within the 20-fold range tested here. Prior studies have shown that sub-neutralizing doses of mixtures of antibodies can select for mutations that increase the avidity of the virus for host cell receptors, as opposed to antigenic mutations within antibody epitopes [36–38]. The range of H17-L19 concentrations tested here is likely above the range of sub-neutralizing concentrations that have been used in the past to select for avidity-enhancing mutations, and it is also possible that mixtures of antibodies targeting different epitopes promote selection for avidity mutants. Understanding the determinants of how a mutation’s differential selection depends on antibody concentration is an interesting area for future work.

Overall, these results confirm that mutational antigenic profiling reproducibly identifies the HA mutations that confer escape from the monoclonal antibody H17-L19. The identified sites of selection are robust across replicate virus libraries and concentrations of antibody.

### Complete mapping of escape mutations from four monoclonal antibodies

**Fig 4:**
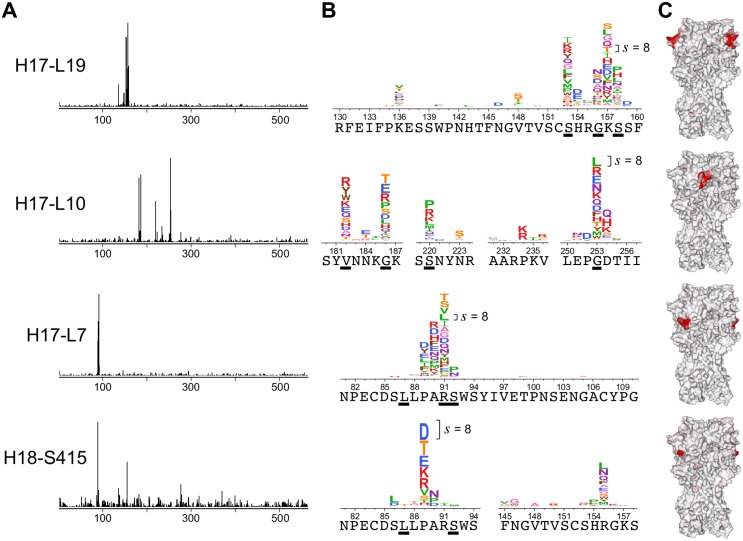
Mutational antigenic profiling of four antibodies. **(A)** Each antibody exerts a different profile of selection on HA. **(B)** Zoomed in view of some of the most strongly selected sites for each antibody. The wild-type amino acid is shown under the logoplots. Sites where mutations were selected in classical escape-mutant selections [11, 12] are underlined. Logoplots spanning all of HA are in S3, S4, S5 and S6 Figs. **(C)** The selection from each antibody visualized on HA’s structure (PDB 1RVX [54]). Each site is colored from white to red based on the differential selection for the most strongly selected mutation at that site. Red indicates strong differential selection. All structures show trimeric HA in the same orientation (the epitope is visible for two of the three monomers for H17-L19, H17-L7, and H18-S415). See S2 Fig for a zoomed-in structural view. The y-axis scale is set separately for each antibody; since the measured strength of differential selection depends on the concentration / potency of the antibody and the mutational tolerance of the viral epitope, it was impossible to precisely standardize selection strength across antibodies. The scale bar in each logo plot shows the letter height for a mutation with differential selection of 8 (a 256-fold enrichment). The data for each antibody is the average across three biological replicates.

We next extended the mutational antigenic profiling to three more antibodies. We performed selections with each antibody at concentrations at which the virus libraries retained 0.1 to 0.4% of their infectivity (S1 Table). Each antibody exerted strong selection at a small number of residues in HA. Fig 4A shows site differential selection across HA, while Fig 4B uses logo plots to show detailed mutation-level selection at some key positions in the antibody epitopes. We again performed three full biological replicates with each antibody, and the results were again highly reproducible among replicates (S1 Fig).

For each antibody, the sites of strongest differential selection were clustered in surface-exposed patches on HA’s structure that are presumably within the antibody-binding footprint (Fig 4C and S2 Fig). The four antibodies target three antigenic regions: H17-L19 targets Ca2, H17-L10 targets Ca1, and H17-L7 and H18-S415 both target Cb [11, 12]. As expected, H17-L19 and H17-L10 exert strong selection on entirely distinct sets of residues, but H17-L7 and H18-S415 exert selection on similar sets of residues in the Cb antigenic region. For three of the antibodies, the strongly selected residues are within short contiguous stretches of primary amino-acid sequence, but for H17-L10 the strongly selected residues are distributed across 70 residues of HA’s primary sequence. Overall, these results show that mutational antigenic profiling can comprehensively identify the selection imposed by diverse antibodies.

### Comparison to traditional neutralization assays

The results above were obtained using experiments that examined tens of thousands of viral variants in parallel. How do these high-throughput measurements compare to the antigenic effects of mutations measured by traditional low-throughput methods? To address this question, we tested some of our key findings with neutralization assays on individual viral mutants. To do this, we used site-directed mutagenesis to introduce single amino-acid mutations into the HA gene, generated viruses by reverse genetics, and performed GFP-based neutralization assays [39].

**Fig 5:**
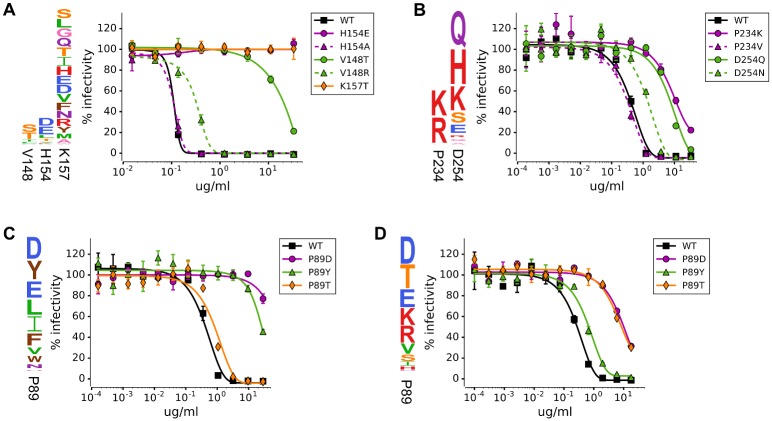
Comparison of the selection measured by mutational antigenic profiling with the antigenic effects of mutations in traditional neutralization assays on individual viral mutants. In each panel, the logo plot shows the results of the mutational antigenic profiling at the sites of mutations chosen for testing, and the graph shows the results of the neutralization assays. There is excellent concordance between whether a mutation is strongly selected in the mutational antigenic profiling and whether it has an effect in the neutralization assay. In many cases, only some of the amino-acid mutations at a site strongly affect neutralization by a given antibody—and the mutational antigenic profiling reliably distinguishes between mutations that do and do not have an effect. The antibodies in each panel are: **(A)** H17-L19, **(B)** H17-L10, **(C)** H17-L7, **(D)** H18-S415.

A clear observation from the mutational antigenic profiling is that at some residues, only a few of the possible amino-acid mutations are strongly selected by any given antibody, concordant with prior work showing that a limited number of mutations are sufficient for antigenic drift [40]. For instance, at HA residue 154, the H17-L19 antibody exerts strong selection only for mutations H154E and H154D, both of which introduce a negatively charged amino acid (Figs 4B and 5A; residues are numbered sequentially beginning at the N-terminal methionine, other numbering schemes are in S1 File). We generated viruses carrying the H154E mutation or a mutation to alanine (H154A), which mutational antigenic profiling did not find to be under differential selection. Neutralization assays confirmed that the H154E mutant completely escaped at all antibody concentrations tested, while the H154A mutant was as sensitive to antibody as wild-type (Fig 5A). Therefore, a more limited method such as alanine scanning would not have identified residue 154 as a site of escape mutations. This finding demonstrates the importance of assaying all amino-acid mutations if the goal is to comprehensively map sites of escape.

Another example of mutation-level sensitivity is HA residue 148, where antibody H17-L19 only selects for mutations to serine and threonine (Fig 4B). Both the V148T and V148S mutations introduce a motif (N-X-S/T) that potentially leads to glycosylation of the asparagine at site 146. To confirm that only some mutations at site 148 enable escape, we generated the V148T mutant as well as another mutant (V148R) that does not introduce a glycosylation motif. As expected, V148T dramatically reduced the virus’s sensitivity to the antibody, whereas V148R only had a small effect (Fig 5A).

The mutational antigenic profiling suggests similar mutation-level sensitivity in escape from antibody H17-L10. At residue 234, there is strong differential selection only for mutations to the positively charged amino-acid residues lysine and arginine (Fig 4B). We generated a virus carrying one of these mutations (P234K) as well as a virus carrying another mutation at the same residue (P234V) that was not under differential selection. Neutralization assays confirmed that the P234K mutation escaped H17-L10, while the P234V mutation caused no change in antibody sensitivity (Fig 5B). Interestingly, in HA’s structure, site 234 is on a neighboring protomer relative to all the other mutations strongly selected by H17-L10 (S2 Fig). Our finding that escape mutations from H17-L10 cross the HA trimer interface is consistent with the fact that this antibody only recognizes trimeric HA [41]. Escape mutations at such epitopes are discernible because mutational antigenic profiling uses actual viruses that display intact HA; such conformational epitopes might not be properly displayed in the modified forms of viral glycoproteins often used in other high-throughput methods such as phage and yeast display.

Overall, these results indicate the power of mutational antigenic profiling to map residues where only a few specific amino-acid mutations lead to escape from antibody. Because this approach examines HA in its native context on influenza virions, it can comprehensively map escape mutations even in complex conformational epitopes.

### Unique repertoires of escape mutations from two antibodies targeting the same site in HA

Two of the antibodies used in our study (H17-L7 and H18-S415) target the same antigenic region of HA, with residue 89 under strong selection from both antibodies (Fig 5C and 5D). Do these antibodies select the same or different amino-acid mutations at this residue? The mutational antigenic profiling suggests that both antibodies select mutations to negatively charged amino acids (P89D and P89E; Fig 5C and 5D). However, each antibody also selects a unique set of additional mutations, such as P89Y for H17-L7 and P89T for H18-S415.

We generated viruses containing the P89D, P89Y, or P89T mutations and tested their sensitivity to both antibodies using neutralization assays. In agreement with the mutational antigenic profiling, the P89D mutant escaped both antibodies, but P89Y only escaped from H17-L7 and P89T only escaped from H18-S415 (Fig 5C and 5D). Thus, when two antibodies target the same site, there can be both common and antibody-specific routes of escape. Characterizing antibody escape at the level of protein sites therefore only provides a partial picture of antigenicity. A complete understanding of escape requires consideration of every mutation at every site.

### Comparison to classical escape-mutant selections

The antigenicity of H1 HA was originally characterized in classic experiments that selected individual viral escape mutants with a panel of mouse monoclonal antibodies [11, 12]. These experiments identified a handful of mutations that ablated binding by each antibody (S2 Table and underlined residues in Fig 4B). All four antibodies used in our study are from the original panel used in the classic experiments. We expected that the sites of differential selection identified by mutational antigenic profiling would include the previously identified mutations.

Indeed, there is strong overlap between sites identified by mutational antigenic profiling and sites of mutations selected in the classic experiments (Fig 4B). However, we also identified numerous additional escape mutations at those and other sites. In some cases, the sites of strongest differential selection were not identified at all in the classic experiments. For instance, as shown in Fig 4, the classic escape-mutant selections failed to identify site 157 for H17-L19, site 89 for H17-L7, and site 89 for H18-S415. Differences in the virus strains used (as discussed below) may account for some of these discrepancies. Additionally, it is likely that mutations at these sites were not uncovered in escape-mutant selections because each such selection only finds one mutation, with a strong bias towards those that arise from single-nucleotide changes that are prevalent in the viral stock. In contrast, our approach simultaneously examines all amino-acid point mutations.

The exception to the concordance between mutational antigenic profiling and classic escape-mutant selections is antibody H18-S415 (Fig 4B). The classic selections failed to identify any mutations at site 89 despite the fact that mutational antigenic profiling finds by far the strongest differential selection at this residue. This discrepancy is not due to spurious signal in the mutational antigenic profiling, since Fig 5D validates that mutations at site 89 potently escape H18-S415. Perhaps the stochasticity of escape-mutant selections caused the classic experiments to fail to probe mutations at site 89.

It is worth noting that the differential selection exerted by H18-S415 in our experiments is substantially noisier than the differential selection for the other antibodies (Fig 4A, S1 Fig). In another recent study, H18-S415 selected an escape virus containing both a mutation in HA and a mutation in the neuraminidase (NA) gene that decreased NA protein expression, leading to increased virus avidity for host cell receptors [42]. The selection of avidity-enhancing mutations has been observed in selection of escape viruses using mixtures of antibodies [37, 38], and it is even possible that the H18-S415 hybridoma cell line is not completely monoclonal. Alternatively, it is possible that it is simply more difficult for the virus to escape H18-S415, and so there is more stochastic noise in which mutations appear in our selections.

The mutational antigenic profiling also failed to find strong selection from H18-S415 for some mutations reported in the classic experiments (L87P, S92P, and E132K; see Fig 4B, S2 Table, and S6 Fig). Why were these mutations selected in the classic experiments but not the mutational antigenic profiling? An important point is that the classic experiments used a different virus strain (A/Puerto Rico/8/1934) than the A/WSN/1933 strain used for our mutational antigenic profiling. In order for a mutation to be under differential selection, it must both support viral replication and affect antigenicity. The mutations L87P, S92P, and E132K are all strongly disfavored under simple selection for viral replication in the A/WSN/1933 strain [32], which likely explains why they are not under strong differential selection in our mutational antigenic profiling. This fact is an important reminder that while mutational antigenic profiling completely maps antibody selection on all single amino-acid mutations that support viral replication in a given viral strain, it does not reveal how the effects of mutations shift with changes in strain background. It remains an open question how well measurements of the effects of mutations on viral replication [43, 44] and antigenicity [42] can be extrapolated beyond the specific genetic backgrounds tested in the lab.

## Discussion

We have used a new high-throughput approach to completely map the amino-acid mutations that enable influenza virus to escape from four neutralizing antibodies. Our approach is conceptually similar to recent methods that couple deep sequencing with phage or yeast display assays for antibody binding [45–47]. But whereas those methods select for binding to antigens expressed in bacteria or yeast, our approach selects for actual neutralization in the context of replication-competent virus. Our experiments therefore measure a phenotype directly relevant to virus evolution: whether a mutation enables a virus to escape neutralization by an antibody.

Our approach also bears similarities to the classic method of selecting individual viral escape mutants. However, escape-mutant selections rely on the occurrence of *de novo* mutations in a viral stock. Therefore, like evolution itself, such selections are stochastic, and only identify one of potentially many escape mutations. In contrast, our massively parallel experiments simultaneously examine all single amino-acid mutations, thereby minimizing stochasticity and allowing us to completely map antibody selection on all point mutations.

The most striking finding from our work is the exquisite mutation level-sensitivity of antibody escape. For each of the four antibodies, we identified residues in HA where only some of the possible amino-acid mutations conferred escape. In some cases, this mutation-level sensitivity is easy to rationalize: we found examples where escape required mutations that introduce glycosylation motifs or change the charge of the amino-acid sidechain. But in other cases, the effects are not only difficult to rationalize but depend on the antibody. For instance, we identified a residue targeted by two antibodies where a mutation that escaped the first antibody had no effect on the second and vice versa. Previous studies have distinguished between an antibody’s “functional epitope” and physical footprint based on the observation that binding is disrupted by mutations at only some residues that contact the antibody [21–24]. Our findings extend this concept by showing that even within the functional epitope, only certain mutations mediate escape, consistent with the observation that a small number of amino-acid mutations in HA can cause extensive antigenic drift of H3N2 influenza virus [40].

These results underscore the shortcomings of thinking about viral antigenic evolution purely in terms of antigenic sites. For instance, many approaches to forecast and model influenza virus evolution are based on partitioning HA into antigenic and non-antigenic sites [4, 5]. However, our work shows that for any individual antibody, it is important to consider the exact amino-acid mutation as well as the site at which it occurs. Application of mutational antigenic profiling to contemporary viral strains and antibodies will enable the prospective mapping of immune-escape mutations on a vastly more comprehensive scale than previously possible.

## Materials and methods

### Mutant virus libraries

The influenza virus mutant libraries used have been described previously [32]. Briefly, reverse-genetics plasmids [48] encoding HA gene were mutagenized at the codon level using a previously described protocol [49]. These plasmid codon-mutant libraries were used to generate libraries of replication-competent influenza viruses using a helper-virus approach that reduced the bottlenecks associated with standard reverse genetics. The virus libraries were then passaged at low MOI to create a genotype-phenotype link between the HA protein on a virion’s surface and the gene that it carries. The viral titers in these libraries were determined by TCID_50_ (50% tissue culture infectious dose) in MDCK-SIAT1 cells (obtained from Sigma Aldrich). Three fully independent virus libraries were generated beginning with independent plasmid mutant libraries as outlined in Fig 2A. It was these low-MOI passaged virus libraries [32] that formed the starting point for the antibody selections described in the current work.

### Antibodies

The antibodies used in this study were originally isolated from mice [11, 12]. Note that in these older papers, two different naming schemes are used for the same antibodies: H17-L19 was also called Ca3; H17-L10 was also called Ca6; H17-L7 was also called Cb15; H18-S415 was also called Cb5. Antibodies secreted by H17-L19, H17-L10, H17-L7, and H18-S415 hybridoma cell lines were purified using PureProteome A/G coated magnetic beads (Millipore). The hybridomas were originally derived from mice at the Wistar Institute [11], and were provided for this study by Scott Hensley.

### Mutant virus selections with antibody

For the selections outlined in Fig 1, we began by diluting each virus library in influenza growth media (Opti-MEM supplemented with 0.01% heat-inactivated FBS, 0.3% BSA, 100 U of penicillin/ml, 100 *μ*g of streptomycin/ml, and 100 *μ*g of calcium chloride/ml) to a concentration of 1 × 10^6^ TCID_50_ per ml. Monoclonal antibody was also diluted in influenza growth media to a concentration twice that intended for use the selection. The virus library was then neutralized by mixing 1 ml of diluted virus with 1 ml of diluted antibody to give the final antibody concentrations listed in S1 Table. This virus-antibody mixture was then incubated at 37°C for 1.5 hours. No-antibody controls were “mock-neutralized” in parallel by substituting influenza growth media for the diluted antibody. At the same time, serial ten-fold dilutions of mutant virus library were made from the 1 × 10^6^ TCID_50_ per ml virus stock to be used as a standard curve to measure infectivity. These dilutions represented 10%, 1%, 0.1%, 0.01%, and 0.001% of the 1 × 10^6^ TCID_50_ dose of library used in neutralizations.

The viral samples were then added to cells to allow infection by non-neutralized virions. We used MDCK-SIAT1 cells that had been plated four hours prior to infection in D10 media (DMEM supplemented with 10% heat-inactivated FBS, 2 mM L-glutamine, 100 U of penicillin/ml, and 100 *μ*g of streptomycin/ml) at 2.5 × 10^5^ cells per well in 6-well dishes. For the infections, we aspirated off the existing D10 media and added the 2 ml of viral sample. Duplicate infections were used for each point on the standard curve of serially diluted virus. After two hours, media in each well was then changed to 2 ml WSN growth media (Opti-MEM supplemented with 0.5% heat-inactivated FBS, 0.3% BSA, 100 U of penicillin/ml, 100 *μ*g of streptomycin/ml, and 100 *μ*g of calcium chloride/ml) after rinsing cells once with PBS to remove residual virus in the supernatant.

Twelve hours later, RNA was isolated from the cells in each well using a Qiagen RNEasy Plus Mini kit by aspirating media, adding 350 *μ*l buffer RLT freshly supplemented with *β*-mercaptoethanol, slowly pipetting several times to lyse cells, transferring the lysate to a RNase-free microfuge tube, vortexing for 20 seconds to homogenize, and proceeding with the manufacturer’s suggested protocol, eluting in 35 *μ*l of RNase-free water.

We estimated the percent remaining infectivity in the neutralized samples using qRT-PCR and a standard curve created using the infections with 10-fold serial dilutions of the virus libraries to give the estimates in S1 Table. For the qPCR, primers WSN-NP-qPCR-F (5’-GCAACGGCTGGTCTGACTCACA-3’) and WSN-NP-qPCR-R (5’-TCCATTCCTGTGCGAACAAG-3’) were used to amplify influenza nucleoprotein (NP) to quantify viral infectivity, and primers 5’-canineGAPDH (5’-AAGAAGGTGGTGAAGCAGGC-3’) and 3’-canineGAPDH (5’-TCCACCACCCTGTTGCTGTA-3’) were used to quantify canine GAPDH to correct for small differences in total RNA amounts. qRT-PCR was performed using Applied Biosystems PowerSYBR green RNA-to-Ct 1-step kit, with 40 ng of RNA in each 20 *μ*l reaction, cycling conditions of 48°C for 30 minutes, 95°C for 10 minutes, and 40 cycles of: 95°C for 15 sec, 58°C for 1 min with data acquisition. All samples were measured in duplicate, and each assay included no-reverse-transcriptase controls. Linear regression of the relationship between the log(infectious dose) and the mean difference in Ct between NP and GAPDH was used to interpolate the remaining infectious dose of each antibody-neutralized sample, expressed as a percentage of the 1 × 10^6^ TCID_50_ used in each neutralization.

### Deep sequencing and quantification of mutation frequencies

To prepare deep sequencing libraries, HA genes were amplified from the RNA isolated from infected cells by reverse transcription with AccuScript Reverse Transcriptase (Agilent 200820) using HA-specific primers WSN-HA-for (5’-AGCAAAAGCAGGGGAAAATAAAAACAAC-3’) and WSN-HA-rev (5’-AGTAGAAACAAGGGTGT TTTTCCTTATATTTCTG-3’). PCR amplification of HA cDNA and Illumina sequencing library preparation was then carried out using a previously described barcoded subamplicon sequencing protocol [32], which was in turn inspired by the approach of Wu and coworkers [33]. The only change made to the previous protocol [32] was that in order to more effectively spread sequencing depth across samples based on the expected diversity of mutations in each sample, the number of uniquely-barcoded single stranded variants used as template for round 2 PCR was 5 × 10^5^ to 7 × 10^5^ for the no-antibody control samples, and 1.5 × 10^5^ for the antibody-neutralized samples. Sequencing libraries with unique indices for each experimental sample were pooled and sequenced on an Illumina HiSeq2500 using 2 x 250 bp paired-end reads in rapid-run mode. S3 Table provides summary statistics of the deep sequencing libraries.

The frequency of each mutation in each sample was determined by using dms_tools [50] (http://jbloomlab.github.io/dms_tools/), version 1.1.20, to align subamplicon reads to a reference HA sequence, group barcodes to build consensus sequences, and quantify mutation counts at every site in the gene for each experimental sample.

### Computation of differential selection

We computed the extent that each mutation is enriched by each antibody selection by comparing mutation counts in each antibody-treated sample to mutation counts from the matching no-antibody control sample, also utilizing controls to account for PCR and sequencing errors. Specifically, we compute the *differential selection* on each mutation as follows. The error rate *ϵ*_*r*,*x*_ at each site *r* for codon *x* is estimated from the apparent frequency of that mutation in our previously described sequencing of HA from wild-type plasmid using barcoded-subamplicon Illumina sequencing [32]. Specifically, the error rate was calculated as:

$$\epsilon_{r,x}=\left(n_{r,x}^{\text{err}}\right)\;/\;\left(\sum_{y}n_{r,y}^{\text{err}}\right)$$ (1)

where $n_{r,x}^{err}$ is the number of counts of codon *x* at site *r* in the wild-type plasmid sequencing library. Note that for the wildtype codon *x* = wt(*r*), *ϵ*_*r*,wt(*r*)_ does not represent the rate of “errors” to this codon, but rather the fraction of reads that give the wildtype codon as expected. We then adjusted the observed counts $n_{r,x}^{\text{mock}}$ and $n_{r,x}^{\text{selected}}$ for codon *x* at site *r* in the mock selected and antibody selected samples, respectively, to the error-corrected counts ${\hat{n}}_{r,x}$ for each sample:

$${\hat{n}}_{r,x}=\left\{\begin{array}{cc} \max\;\left[\left(\sum_{y}n_{r,y}\right)\;\left(\frac{n_{r,x}}{\sum_{y}n_{r,y}}-\epsilon_{r,x}\right),0\right] & \text{if}\;x\neq \mathrm{wt}\left(r\right)\\ n_{r,x}/\epsilon_{r,x} & \text{if}\;x=\mathrm{wt}\left(r\right). \end{array}\right.$$ (2)

This correction ignores second-order terms in which a mutant codon is incorrectly read as another mutant codon or wildtype due to sequencing errors; however, provided that both error rates and mutation rates are low (which is the case in our experiments), these second-order terms can be safely ignored.

To convert from codon counts to amino-acid counts, we summed the error-adjusted counts for all codons encoding each amino acid *a* at site *r* to give the error-adjusted amino-acid counts ${\hat{n}}_{r,a}^{\text{mock}}$ and ${\hat{n}}_{r,a}^{\text{selected}}$ for the mock selected and antibody selected samples, respectively. We then computed the relative enrichment *E*_*r*,*a*_ of amino acid *a* at site *r* as

$$E_{r,a}=\frac{\left({\hat{n}}_{r,a}^{\text{selected}}+f_{r,\text{selected}}\times P\right)/\left({\hat{n}}_{r,\mathrm{wt}\left(r\right)}^{\text{selected}}+f_{r,\text{selected}}\times P\right)}{\left({\hat{n}}_{r,a}^{\text{mock}}+f_{r,\text{mock}}\times P\right)/\left({\hat{n}}_{r,\mathrm{wt}\left(r\right)}^{\text{mock}}+f_{r,\text{mock}}\times P\right)}$$ (3)

where wt(*r*) denotes the wildtype amino acid at site *r*, *P* is a pseudocount (set to 10 in our analyses), and $f_{r,\text{selected}}$ and $f_{r,\text{mock}}$ give the relative depths of the selected and mock samples at site *r*:

$$f_{r,\text{selected}}=\max\;\left[1,\left(\sum_{a}n_{r,a}^{\text{selected}}\right)\;/\;\left(\sum_{a}n_{r,a}^{\text{mock}}\right)\right]$$ (4)

$$f_{r,\text{mock}}=\max\;\left[1,\left(\sum_{a}n_{r,a}^{\text{mock}}\right)\;/\;\left(\sum_{a}n_{r,a}^{\text{selected}}\right)\right]$$ (5)

The reason for scaling the pseudocount by the library depth is that in the absence of such scaling, if the selected and mock samples are sequenced at different depths, the estimates of *E*_*r*,*a*_ will tend to be systematically different from one even if the relative counts are the same in both conditions.

The mutation differential selection values are the logarithm of the enrichment values:

$$s_{r,a}=\log_{2}\;E_{r,a}.$$ (6)

Mutations that confer escape from an antibody will have a larger relative frequency in the antibody-selected sample than the no-antibody control sample, and will thus have a large, positive differential selection. Therefore, we limited analysis to positive differential selection to identify antibody escape mutations. To summarize the differential selection at each site, we sum the mutation differential selection values *s*_*r*,*a*_ over all amino-acids *a* with positive mutation differential selection and term this the positive site differential selection *s*_*r*_ for site *r*:

$$s_{r}=\sum_{a}\max\;\left(0,s_{r,a}\right).$$ (7)

Logoplots visualizing differential selection display each amino acid with a height proportional to the mutation differential selection *s*_*r*,*a*_. Amino acid letter codes are colored based on the physiochemical properties of the amino-acid side chain: hydrophobic (V, L, I, M, P) are green, nucleophilic (S, T, C) are orange, small (A, G) are pink, aromatic (F, Y, W) are brown, amide (N, Q) are purple, positively-charged (H, K, R) are red, and negatively-charged (D, E) are blue.

The computer code to perform these differential selection analyses is incorporated in the dms_tools (http://jbloomlab.github.io/dms_tools/) software as the program dms_diffselection. The logoplots created by dms_tools are rendered with WebLogo [51].

### GFP-based neutralization assays

We performed neutralization assays using viruses carrying GFP in the PB1 segment using a previously described protocol [39]. These GFP reporter viruses were generated using seven bidirectional reverse genetics plasmids [52] encoding the PB2, PA, HA, NP, NA, M, and NS segments of A/WSN/1933 (kindly provided by Robert Webster of St. Jude Children’s Research Hospital), and a unidirectional reverse genetics plasmid pHH-PB1flank-GFP in which the coding sequence of PB1 is replaced by GFP [53]. Since these viruses carry GFP instead of PB1, they are grown in complementing 293T-CMV-PB1 (derived from cells purchased from the American Typed Culture Collection as described in [53]) and MDCK-SIAT1-CMV-PB1 cells (derived from cells purchased from Sigma Aldrich as described in [53]) that constitutively express the WSN PB1 protein.

For each HA mutation tested in the neutralization assay, the indicated amino-acid mutation was introduced into the WSN HA bidirectional reverse genetics plasmid by site-directed mutagenesis, and the HA sequence was verified by Sanger sequencing. To generate each mutant GFP-carrying virus, we transfected a co-culture of 293T-CMV-PB1 and MDCK-SIAT1-CMV-PB1 cells with the eight reverse genetics plasmids described above. For each transfection, 4 × 10^5^ 293T-CMV-PB1 and 4 × 10^4^ MDCK-SIAT1-CMV-PB1 per well were plated in 6-well plates in D10 media four hours prior to transfection. Each well received a transfection mixture of 100 *μ*l DMEM, 3 *μ*l BioT transfection reagent, and 250 ng of each of the eight reverse genetics plasmids. At 20 hours post-transfection, the media was changed to WSN neutralization media, which has low autofluorescence in the GFP channel (Medium 199 supplemented with 0.3% BSA, 100 U of penicillin/ml, 100 g of streptomycin/ml, 100 g of calcium chloride/ml, 25 mM HEPES, 0.5% FBS). At 72 hours post-transfection, culture supernatants were clarified by centrifugation at 2,000×g, aliquoted, and frozen at -80°C.

The GFP-carrying viruses were titered by flow cytometry in MDCK-SIAT1-CMV-PB1 cells. For this titering, cells were plated in 12-well plates at 1 × 10^5^ cells per well in WSN neutralization media. Four hours after plating, cells were infected with dilutions of viral supernatant. At 16 hours after infection, wells with approximately 1% of cells GFP-positive were analyzed by flow cytometry, and the fraction of GFP-positive cells was used to calculate the titer of infectious particles in each viral supernatant.

For the neutralization assays, monoclonal antibody was diluted down columns of a 96-well plate in WSN neutralization media. Three replicate dilution columns were used for each virus-antibody combination. Columns without antibody were used to measure maximal fluorescence in the absence of neutralization, and columns without cells were used to measure background fluorescence in viral supernatants, which we found to contribute more background fluorescence than cells alone. The GFP reporter viruses were diluted in WSN neutralization media to 1 × 10^3^ infectious particles per *μ*l and 40 *μ*l (4 × 10^4^ infectious particles) was added to each well. Plates were incubated at 37°C for 1.5 hours before adding 4 × 10^4^ MDCK-SIAT1-CMV-PB1 cells to each well. After 16 hours incubation at 37°C, GFP fluorescence intensity was measured on a Tecan plate reader using an excitation wavelength of 485 nm and an emission wavelength of 515 nm (12-nm slit widths). Percent of maximal infectivity was calculated by subtracting background fluorescence signal from all wells and dividing the signal from antibody-containing wells by the signal from corresponding wells without antibody.

## Supporting information

S1 Fig Positive site differential selection is highly correlated between full biological replicate measurements on independently generated mutant virus libraries. Shown are correlations in positive site differential selection for antibodies **(A)** H17-L10, **(B)** H17-L7, and **(C)** H18-S415. Correlation coefficients are Pearson’s R.
(TIF)

S2 Fig Detailed view of differential selection by each antibody projected onto HA’s structure. Each panel zooms into the relevant region of the structure shown in Fig 4C for that antibody. Residues are colored from white to red based on the differential selection for the most strongly selected mutation at that site for each antibody. Asterisks mark sites of strong differential selection which were not found in the original antigenic mapping of HA with that antibody [11, 12]. **(A)** H17-L19. **(B)** H17-L10. Strong differential selection at site 223 (not visible) results in putative glycosylation at site 221. The dashed line marks the boundary between two adjacent HA protomers. **(C)** H17-L7. **(D)** H18-S415.
(TIF)

S3 Fig A logo plot showing the differential selection across all of HA from antibody H17-L19 at the concentration used in Fig 4. These data are the average across the replicate libraries for each antibody.
(PDF)

S4 Fig A logo plot showing the differential selection across all of HA from antibody H17-L10 at the concentration used in Fig 4. These data are the average across the replicate libraries for each antibody.
(PDF)

S5 Fig A logo plot showing the differential selection across all of HA from antibody H17-L7 at the concentration used in Fig 4. These data are the average across the replicate libraries for each antibody.
(PDF)

S6 Fig A logo plot showing the differential selection across all of HA from antibody H18-S415 at the concentration used in Fig 4. These data are the average across the replicate libraries for each antibody.
(PDF)

S1 File HA numbering scheme. This text file shows the sequence and sequential A/WSN/1933 HA numbering scheme used in this study. It also gives the corresponding numbers in the 1RVX PBD HA structure and in an H3 numbering system.
(TXT)

S2 File Computer code. This ZIP archive contains the computer code to perform the data analysis described in this manuscript. The analysis is performed by the iPython notebook found in the ZIP archive.
(ZIP)

S3 File Mutation differential selection values. This ZIP archive contains text files giving the numerical mutation differential selection values for each antibody at each concentration tested. These values are the average across the replicate libraries for each condition
(ZIP)

S1 Table Percentage of each mutant virus library remaining infectious after antibody neutralization in each replicate selection experiment. Percent infectivity was measured by qRT-PCR of the influenza nucleoprotein gene and interpolated from a standard curve of infection prepared with serial dilutions of each virus library.
(PDF)

S2 Table All mutations identified in the classic escape mutant selections with the four antibodies used in our study. Note that the classic experiments used the A/Puerto Rico/8/1934 (H1N1) virus, whereas our study used the A/WSN/1933 (H1N1) virus. In the older papers, multiple names were used to refer to the same antibody: H17-L19 was also called Ca3; H17-L10 was also called Ca6; H17-L7 was also called Cb15; H18-S415 was also called Cb5.
(PDF)

S3 Table Summary statistics for barcoded subamplicon sequencing libraries. Sample names designate the mutant virus library used (L1, L2, or L3), the antibody used for selection (or mock in absence of antibody), the relative antibody concentration used if applicable (c1 is lowest, c3 is highest), and technical replicate (r1 or r2) if applicable. Targeted subamplicon barcode complexity refers to the number of uniquely barcoded molecules used in round 2 PCR (see methods) for each of the six HA subamplicons. Targeted total barcode complexity accounts for all six HA subamplicons. Total reads is the total number of paired-end sequencing reads obtained. Total aligned barcodes is the total number of barcodes (across all six subamplicons) that could be aligned with at least two paired-end sequencing reads. Median effective depth is the median number of barcodes aligned per HA codon. Previous deep sequencing of the input libraries used here [32] found at least three occurrences of between 47% and 51% of the total possible amino-acid mutations (over 97% of the possible amino-acid mutations were found at least three times in the starting plasmid mutant libraries before functional selection removed mutations incompatible with viral growth).
(PDF)
