# Supplementary figures and images for: Complete mapping of viral escape from neutralizing antibodies
Source: PLoS Pathog. 2017 Mar 13;13(3):e1006271. doi: 10.1371/journal.ppat.1006271 (PMC5363992; doi:10.1371/journal.ppat.1006271)

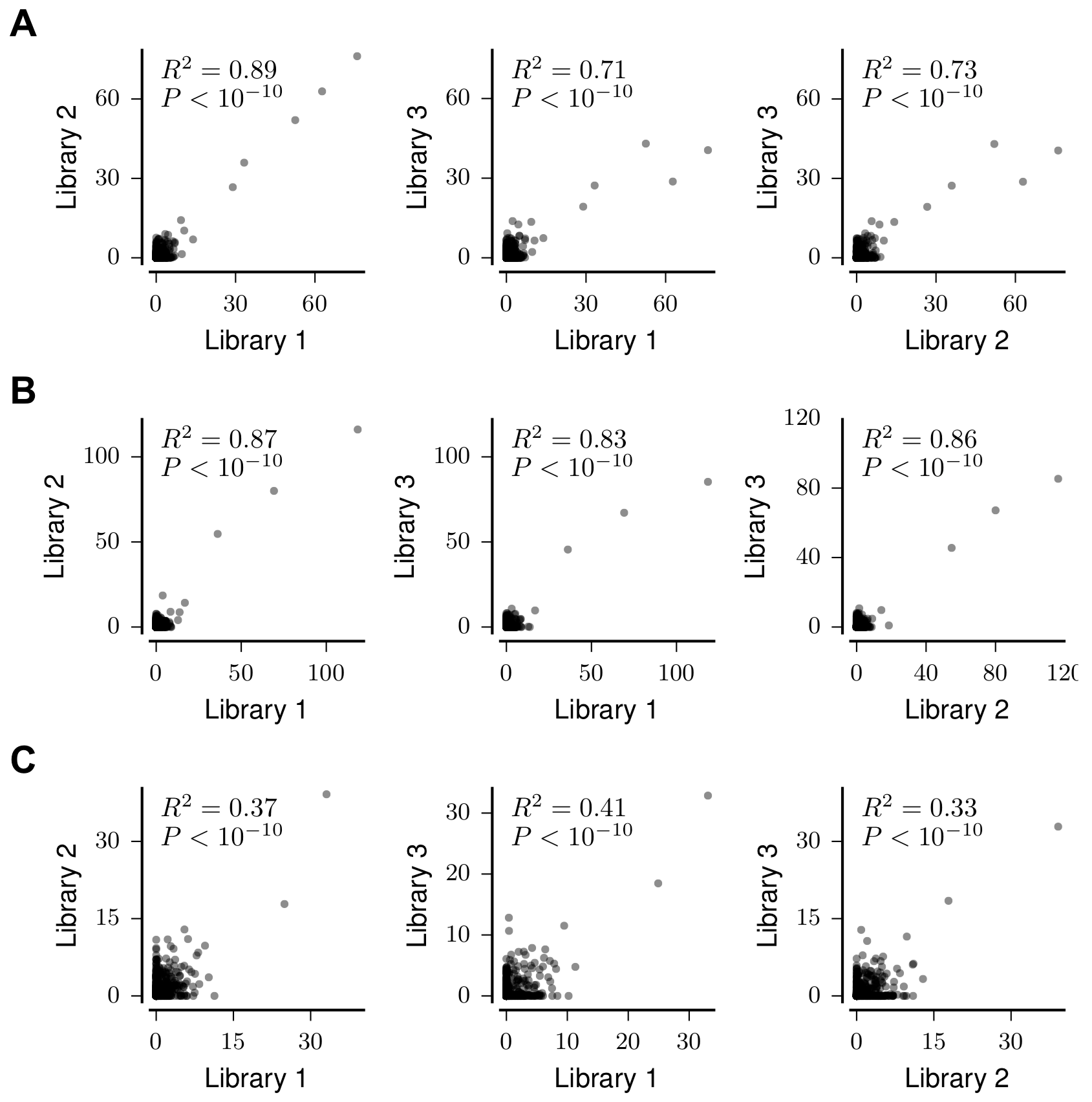

Supplement: S1 Fig — Shown are correlations in positive site differential selection for antibodies (A) H17-L10, (B) H17-L7, and (C) H18-S415. Correlation coefficients are Pearson’s R. (TIF) [file ppat.1006271.s001.tif]

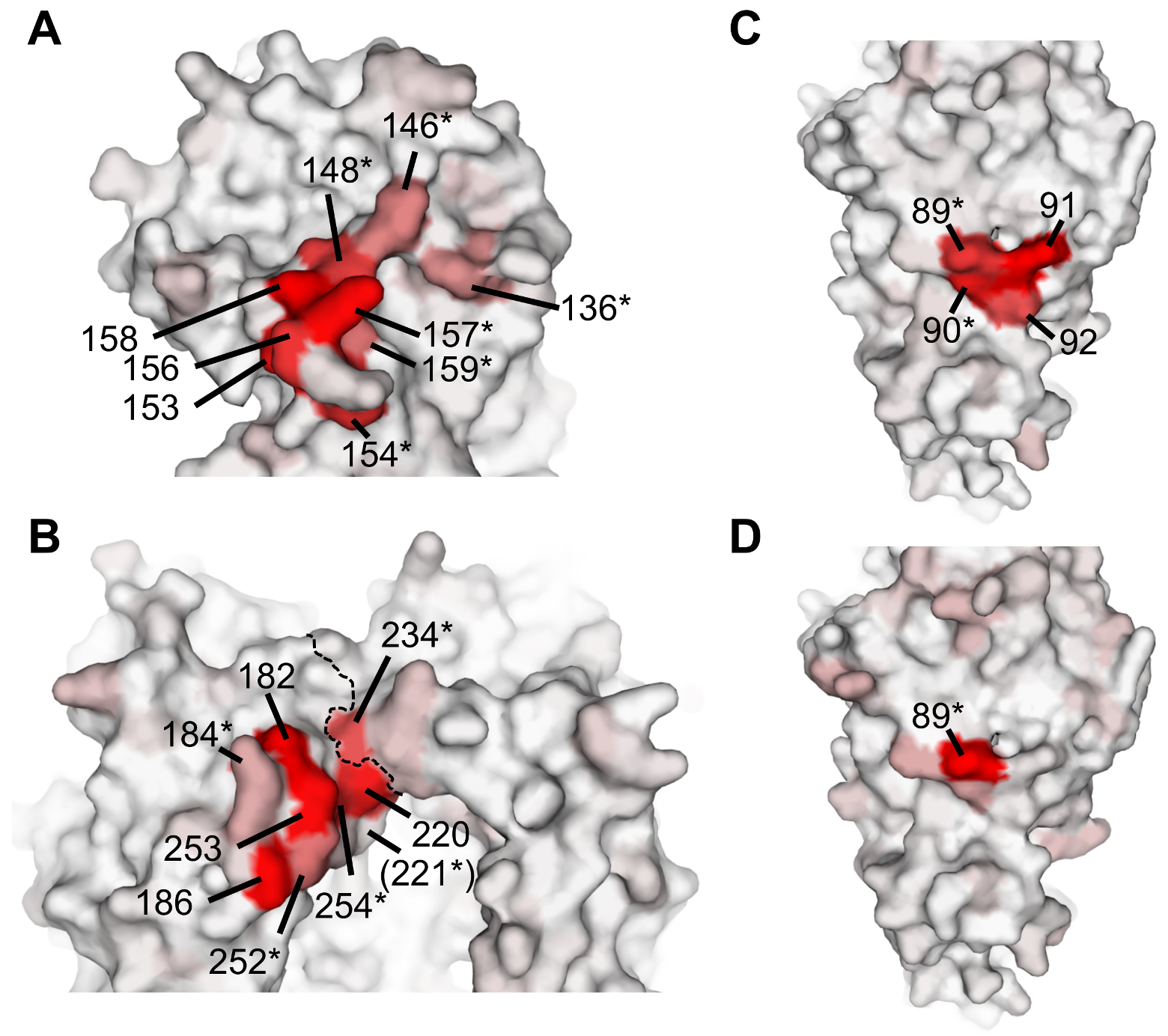

Supplement: S2 Fig — Each panel zooms into the relevant region of the structure shown in Fig 4C for that antibody. Residues are colored from white to red based on the differential selection for the most strongly selected mutation at that site for each antibody. Asterisks mark sites of strong differential selection which were not found in the original antigenic mapping of HA with that antibody [11, 12]. (A) H17-L19. (B) H17-L10. Strong differential selection at site 223 (not visible) results in putative glycosylation at site 221. The dashed line marks the boundary between two adjacent HA protomers. (C) H17-L7. (D) H18-S415. (TIF) [file ppat.1006271.s002.tif]

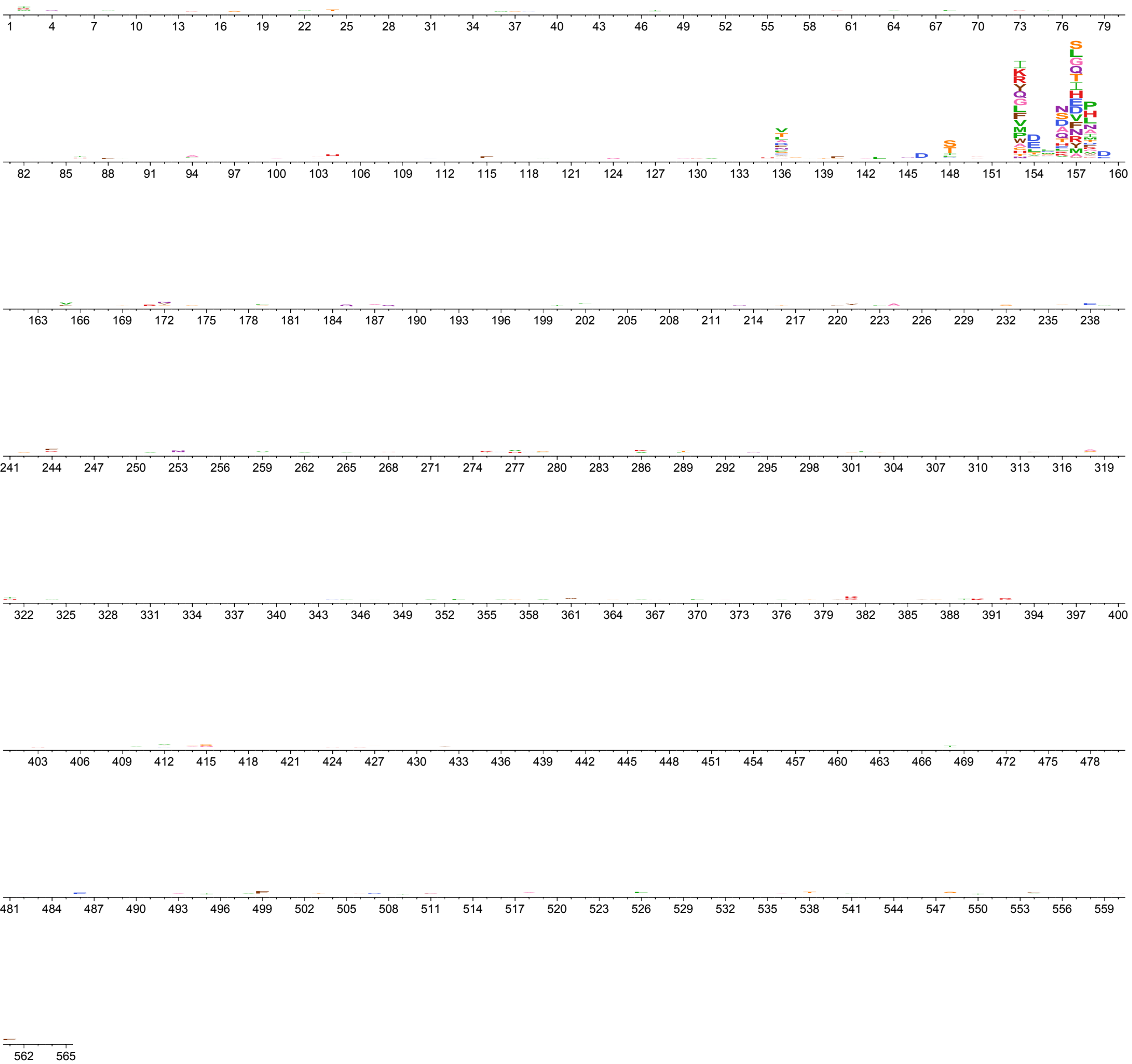

Supplement: S3 Fig — These data are the average across the replicate libraries for each antibody. (PDF) [file ppat.1006271.s003.pdf]

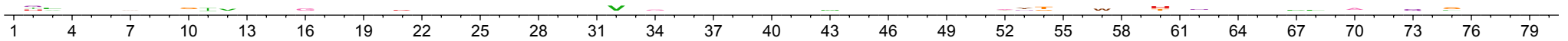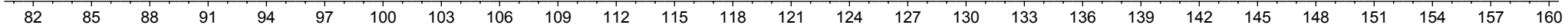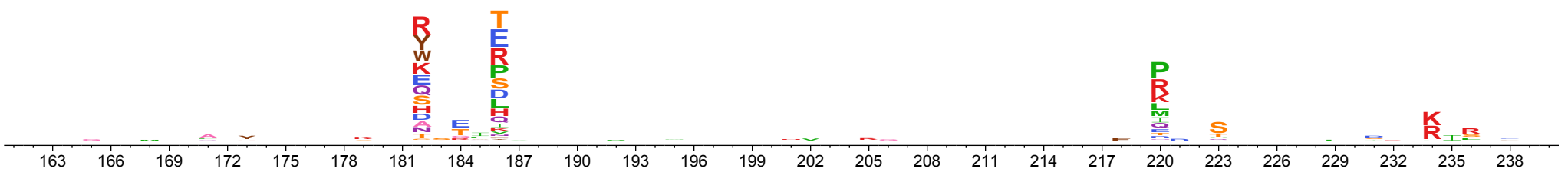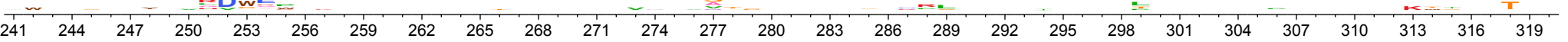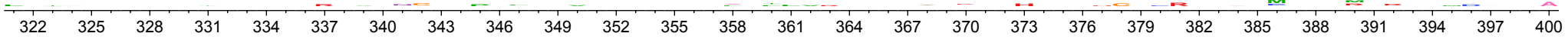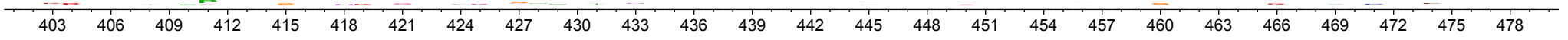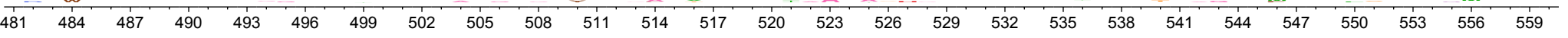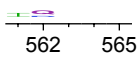

Supplement: S4 Fig — These data are the average across the replicate libraries for each antibody. (PDF) [file ppat.1006271.s004.pdf]

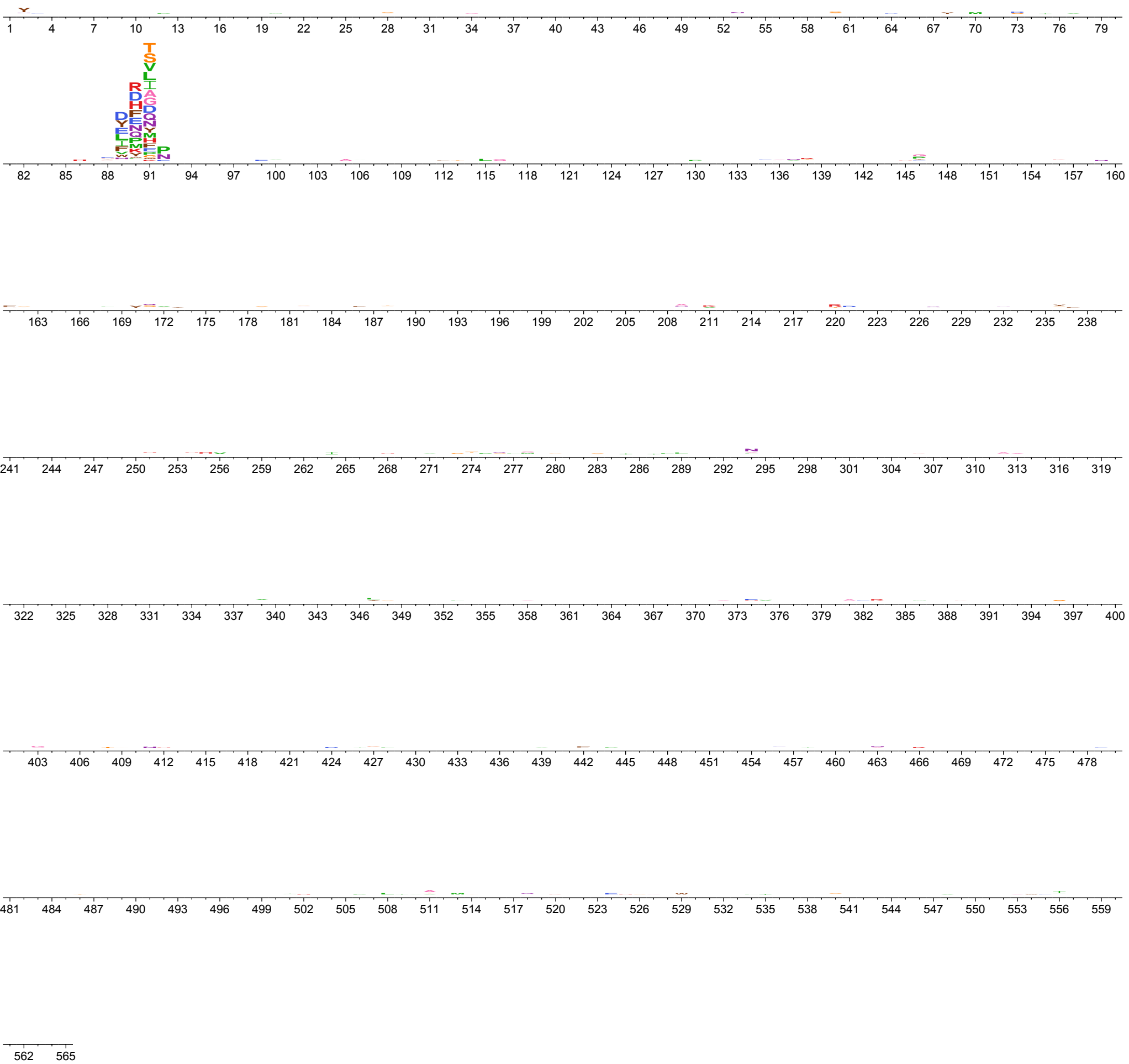

Supplement: S5 Fig — These data are the average across the replicate libraries for each antibody. (PDF) [file ppat.1006271.s005.pdf]

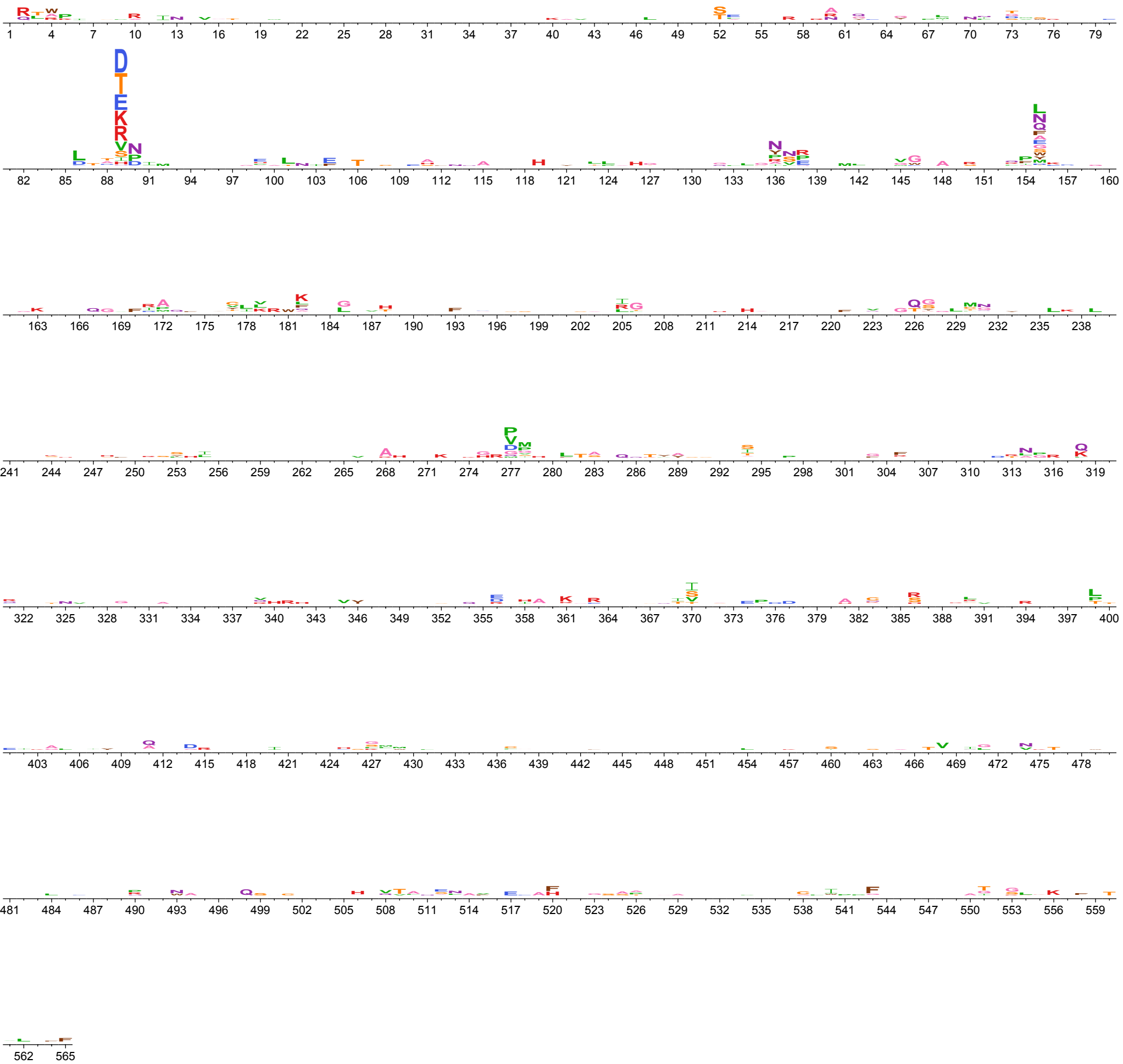

Supplement: S6 Fig — These data are the average across the replicate libraries for each antibody. (PDF) [file ppat.1006271.s006.pdf]
