# Supplementary material for: Complete mapping of viral escape from neutralizing antibodies
Source: PLoS Pathog. 2017 Mar 13;13(3):e1006271. doi: 10.1371/journal.ppat.1006271 (PMC5363992; doi:10.1371/journal.ppat.1006271)
Supplement: S2 File — This ZIP archive contains the computer code to perform the data analysis described in this manuscript. The analysis is performed by the iPython notebook found in the ZIP archive. (ZIP) [file ppat.1006271.s008.zip › S2_File/SequencingAnalysisAndFigures.html]

SequencingAnalysisAndFigures


# Analysis of monoclonal antibody selection of mutant virus libraries of A/WSN/1933(H1N1) hemagglutinin¶

Mike Doud, 2016

## import modules and define variables¶

In [1]:

```
import os
import glob
import subprocess
import time
import pandas as pd
import numpy as np
import matplotlib
print "Using matplotlib version %s" % matplotlib.__version__
matplotlib.use("Pdf")
from matplotlib.ticker import MaxNLocator
import pylab as plt
from colour import Color
from IPython.display import Image, display
import dms_tools
print("Using dms_tools version {0}".format(dms_tools.__version__))

def ShowPDF(pdfs, width=None):
    '''Displays images in *pdfs*, which can be one PDF or list of PDFs. Multiple images displayed side-by-side.'''
    png = '_temp.png'
    if not isinstance(pdfs, list):
        pdfs = [pdfs]
    subprocess.check_call(['convert', '-density', '134', '-trim'] + pdfs + ['+append', png])
    time.sleep(0.5)
    display(Image(png, width=width))

# make directories for the analysis
dirs = {'fastq':'./FASTQ_files',
        'counts':'./counts_and_alignments/',
        'diffsel':'./differential_selections/',
        'plots':'./plots'}
for xdir in dirs.values():
    if not os.path.isdir(xdir):
        os.mkdir(xdir)

refseq = 'WSN_HA_reference.fa' # WSN HA nucleotide sequence used in these experiments
errorcontrolcounts = 'err_counts.txt' # counts for wildtype DNA from Doud and Bloom, 2016, Viruses
alignspecs = '1,285,36,37 286,570,31,32 571,855,37,32 856,1140,31,36 1141,1425,29,33 1426,1698,40,43' # see docs for `dms_barcodedsubamplicons`
```

```
Using matplotlib version 1.5.3
Using dms_tools version 1.1.20
```

## define experimental samples and download sequencing data from the SRA¶

this downloads the `.SRA` file for each sample and then uses `fastq-dump` to extract `FASTQ` files for read 1 and read 2.

In [2]:

```
# sample names are structured as `LX_mAb_cX_rX` where LX denotes the mutant virus *L*ibrary used, mAb indicates the antibody used, 
# and cX and rX indicate different antibody concentrations or technical replicates when they exist. `mock` is no-antibody control.
SRA_samples = {
    'SRR4841569':'L1_H17L19_c1_r1',
    'SRR4841570':'L1_H17L19_c2_r1',
    'SRR4841567':'L1_H17L19_c3_r1',
    'SRR4841568':'L1_H17L19_c1_r2',
    'SRR4841573':'L1_H17L19_c2_r2',
    'SRR4841574':'L1_H17L19_c3_r2',
    'SRR4841571':'L2_H17L19_c1',
    'SRR4841572':'L2_H17L19_c2',
    'SRR4841575':'L2_H17L19_c3',
    'SRR4841576':'L3_H17L19_c1',
    'SRR4841595':'L3_H17L19_c2',
    'SRR4841596':'L3_H17L19_c3',
    'SRR4841597':'L1_H17L10',
    'SRR4841598':'L2_H17L10',
    'SRR4841599':'L3_H17L10',
    'SRR4841600':'L1_H17L7',
    'SRR4841601':'L2_H17L7',
    'SRR4841602':'L3_H17L7',
    'SRR4841603':'L1_H18S415',
    'SRR4841604':'L2_H18S415',
    'SRR4841579':'L3_H18S415',
    'SRR4841578':'L1_mock_r1',
    'SRR4841581':'L1_mock_r2',
    'SRR4841580':'L2_mock',
    'SRR4841582':'L3_mock'}

# A list of experimental sample names, which will be used to iterate over samples throughout the analysis:
samples = SRA_samples.values()

# download sequencing data if it doesn't already exist:
for (accession, samplename) in SRA_samples.items():
    if (os.path.isfile('{0}/{1}_1.fastq'.format(dirs['fastq'], samplename)) and os.path.isfile('{0}/{1}_2.fastq'.format(dirs['fastq'], samplename))):
        print "The FASTQ files for sample {0}, which corresponds to accession {1}, already exist.".format(samplename, accession)
    else:
        print "Downloading and converting file format for sample {0}, which corresponds to accession {1}.".format(samplename, accession)
        !cd FASTQ_files; wget --no-clobber 'ftp://ftp-trace.ncbi.nih.gov/sra/sra-instant/reads/ByRun/sra/SRR/SRR484/'$accession'/'$accession'.sra'
        !mv "FASTQ_files/"$accession".sra" "FASTQ_files/"$samplename".sra"
        !fastq-dump -O FASTQ_files/ --split-files "FASTQ_files/"$samplename".sra"
```

```
The FASTQ files for sample L1_H17L19_c2_r2, which corresponds to accession SRR4841573, already exist.
The FASTQ files for sample L1_mock_r1, which corresponds to accession SRR4841578, already exist.
The FASTQ files for sample L3_H18S415, which corresponds to accession SRR4841579, already exist.
The FASTQ files for sample L3_H17L19_c3, which corresponds to accession SRR4841596, already exist.
The FASTQ files for sample L1_H17L10, which corresponds to accession SRR4841597, already exist.
The FASTQ files for sample L3_H17L19_c2, which corresponds to accession SRR4841595, already exist.
The FASTQ files for sample L1_H17L19_c2_r1, which corresponds to accession SRR4841570, already exist.
The FASTQ files for sample L2_H17L19_c1, which corresponds to accession SRR4841571, already exist.
The FASTQ files for sample L2_H17L10, which corresponds to accession SRR4841598, already exist.
The FASTQ files for sample L3_H17L10, which corresponds to accession SRR4841599, already exist.
The FASTQ files for sample L1_H17L19_c3_r2, which corresponds to accession SRR4841574, already exist.
The FASTQ files for sample L2_H17L19_c2, which corresponds to accession SRR4841572, already exist.
The FASTQ files for sample L3_H17L19_c1, which corresponds to accession SRR4841576, already exist.
The FASTQ files for sample L3_H17L7, which corresponds to accession SRR4841602, already exist.
The FASTQ files for sample L1_H18S415, which corresponds to accession SRR4841603, already exist.
The FASTQ files for sample L1_H17L7, which corresponds to accession SRR4841600, already exist.
The FASTQ files for sample L2_H17L7, which corresponds to accession SRR4841601, already exist.
The FASTQ files for sample L2_H18S415, which corresponds to accession SRR4841604, already exist.
The FASTQ files for sample L2_H17L19_c3, which corresponds to accession SRR4841575, already exist.
The FASTQ files for sample L1_mock_r2, which corresponds to accession SRR4841581, already exist.
The FASTQ files for sample L2_mock, which corresponds to accession SRR4841580, already exist.
The FASTQ files for sample L3_mock, which corresponds to accession SRR4841582, already exist.
The FASTQ files for sample L1_H17L19_c1_r1, which corresponds to accession SRR4841569, already exist.
The FASTQ files for sample L1_H17L19_c1_r2, which corresponds to accession SRR4841568, already exist.
The FASTQ files for sample L1_H17L19_c3_r1, which corresponds to accession SRR4841567, already exist.
```

## compute mutation counts from FASTQ files for each experimental sample¶

See documentation for `dms_barcodedsubamplicons` for details on the algorithm for parsing mutation counts from FASTQ files.

In [3]:

```
for sample in samples:
    r1files = ['{0}/{1}_1.fastq'.format(dirs['fastq'], sample)]
    r2files = ['{0}/{1}_2.fastq'.format(dirs['fastq'], sample)]
    outprefix = '{0}/{1}_'.format(dirs['counts'], sample)
    
    subprocess.check_call(['dms_barcodedsubamplicons',
                           outprefix,
                           refseq,
                           ','.join(r1files),
                           ','.join(r2files)] + 
                           alignspecs.split() + [
                           '--R1trimlength', '200',
                           '--R2trimlength', '170',
                          ])
```

Show some summary statistics for alignments. Break into groups of alignments based on mAb for clarity.

In [4]:

```
lib1_H17L19_samples = [s for s in samples if 'L1_H17L19' in s]
lib23_H17L19_samples = [s for s in samples if 'L2_H17L19' in s or 'L3_H17L19' in s]
H17L7_samples = [s for s in samples if 'H17L7' in s]
H17L10_samples = [s for s in samples if 'H17L10' in s]
H18S415_samples = [s for s in samples if 'H18S415' in s]
mock_samples = [s for s in samples if 'mock' in s] 

for sample_group, group_prefix in zip(
    [lib1_H17L19_samples, lib23_H17L19_samples, H17L7_samples, H17L10_samples, H18S415_samples, mock_samples],
    ['lib1_H17L19_', 'lib23_H17L19_', 'H17L7_', 'H17L10_', 'H18S415_', 'mock_']):
    
    print "here is the summary of the alignments for {0}".format(group_prefix[:-1])
    summary_list = []
    summary_prefix = '{0}/{1}'.format(dirs['counts'], group_prefix)
    for sample in sample_group:
        sample_outprefix = '{0}/{1}_'.format(dirs['counts'], sample) # the outprefix used for this sample by dms_barcodedsubamplicons
        summary_list.append(sample_outprefix + ',' + sample.replace('_','-')) # be sure to replace any underscores in the name of the alignment since they are processed by LaTeX.
    subprocess.check_call(['dms_summarizealignments',
                           summary_prefix,
                           'barcodedsubamplicons'] + 
                           summary_list
                          )
    
    for suffix in ['barcodes.pdf', 'depth.pdf', 'mutdepth.pdf', 'mutfreqs.pdf']:
        ShowPDF(summary_prefix + suffix, width=500)
```

```
here is the summary of the alignments for lib1_H17L19
```

```
here is the summary of the alignments for lib23_H17L19
```

```
here is the summary of the alignments for H17L7
```

```
here is the summary of the alignments for H17L10
```

```
here is the summary of the alignments for H18S415
```

```
here is the summary of the alignments for mock
```

## compute differential selection¶

see documentation for `dms_diffselection` for details on the algorithm to compute differential selection between antibody-neutralized and no-antibody control samples.

In [5]:

```
for mab in ['H17L19', 'H17L10', 'H17L7', 'H18S415']:
    antibody_samples = [s for s in samples if mab in s]
    for antibody_sample in antibody_samples:
        # specify the proper mock control for each antibody sample
        if 'r1' in antibody_sample:
            mock_sample = 'L1_mock_r1'
        elif 'r2' in antibody_sample:
            mock_sample = 'L1_mock_r2'
        elif 'L1_' in antibody_sample: # a library-1 selection that wasn't done in technical replicate; just use one of the replicate mock for library-1.
            mock_sample = 'L1_mock_r1'
        elif 'L2_' in antibody_sample:
            mock_sample = 'L2_mock'
        elif 'L3_' in antibody_sample:
            mock_sample = 'L3_mock'
        else:
            raise ValueError('could not identify which replicate was used in selected sample to assign the proper mock sample.')

        outprefix = "{0}/{1}_vs_{2}_".format(dirs['diffsel'], antibody_sample, mock_sample)
        mock_counts = '{0}/{1}_counts.txt'.format(dirs['counts'], mock_sample)
        antibody_counts = '{0}/{1}_counts.txt'.format(dirs['counts'], antibody_sample)
        
        subprocess.check_call(['dms_diffselection',
                                mock_counts,
                                antibody_counts,
                                outprefix,
                                '--errorcontrolcounts', errorcontrolcounts,
                                ])
```

as a negative control to gauge experimental noise between replicates when there is NO differential selection, compute differential selection between technical replicates of no-antibody selection performed on library 1:

In [6]:

```
subprocess.check_call(['dms_diffselection',
                        '{0}/L1_mock_r1_counts.txt'.format(dirs['counts']),
                        '{0}/L1_mock_r2_counts.txt'.format(dirs['counts']),
                        "{0}/L1_mockr1_vs_mockr2_".format(dirs['diffsel']),
                        '--errorcontrolcounts', errorcontrolcounts,
                        ])
```

Out[6]:

```
0
```

## plot correlations in differential selection between replicates¶

In [7]:

```
def PlotCorrelations(file1, file2, outprefix, name1, name2, markersize=6, alpha=0.45, corr_on_plot=True):
    commands = ['dms_correlate',
                   file1,
                   file2,
                   outprefix,
                   '--name1', name1,
                   '--name2', name2,
                   '--alpha', str(alpha),
                   '--restrictdiffsel', 'positive',
                   '--r2',
                   '--markersize', str(markersize)
                  ]
    if corr_on_plot:
        commands.append('--corr_on_plot')
    subprocess.check_call(commands)
```

technical and biological replicate correlations for positive site differential selection for the highest concentration of H17-L19:

In [8]:

```
L1_tech_reps = ['{0}/L1_H17L19_c3_r1_vs_L1_mock_r1_sitediffsel.txt'.format(dirs['diffsel']),
                '{0}/L1_H17L19_c3_r2_vs_L1_mock_r2_sitediffsel.txt'.format(dirs['diffsel'])]
bio_reps = ['{0}/L1_H17L19_c3_r1_vs_L1_mock_r1_sitediffsel.txt'.format(dirs['diffsel']),
           '{0}/L2_H17L19_c3_vs_L2_mock_sitediffsel.txt'.format(dirs['diffsel']),
           '{0}/L3_H17L19_c3_vs_L3_mock_sitediffsel.txt'.format(dirs['diffsel'])]

plots = []

outfile = '{0}/Corr_H17L19_techreps'.format(dirs['plots'])
plots.append(outfile)
PlotCorrelations(L1_tech_reps[0], L1_tech_reps[1], outfile, 'Library 1', 'Library 1 replicate')

outfile = '{0}/Corr_H17L19_biorep12'.format(dirs['plots'])
plots.append(outfile)
PlotCorrelations(bio_reps[0], bio_reps[1], outfile, 'Library 1', 'Library 2')

outfile = '{0}/Corr_H17L19_biorep13'.format(dirs['plots'])
plots.append(outfile)
PlotCorrelations(bio_reps[0], bio_reps[2], outfile, 'Library 1', 'Library 3')

outfile = '{0}/Corr_H17L19_biorep23'.format(dirs['plots'])
plots.append(outfile)
PlotCorrelations(bio_reps[1], bio_reps[2], outfile, 'Library 2', 'Library 3')

ShowPDF([f + '.pdf' for f in plots])
```

technical and biological replicate correlations for mutation differential selection for the same comparisons:

In [9]:

```
L1_tech_reps = ['{0}/L1_H17L19_c3_r1_vs_L1_mock_r1_mutdiffsel.txt'.format(dirs['diffsel']),
                '{0}/L1_H17L19_c3_r2_vs_L1_mock_r2_mutdiffsel.txt'.format(dirs['diffsel'])]
bio_reps = ['{0}/L1_H17L19_c3_r1_vs_L1_mock_r1_mutdiffsel.txt'.format(dirs['diffsel']),
           '{0}/L2_H17L19_c3_vs_L2_mock_mutdiffsel.txt'.format(dirs['diffsel']),
           '{0}/L3_H17L19_c3_vs_L3_mock_mutdiffsel.txt'.format(dirs['diffsel'])]

plots = []
markersize = 3.5
alpha = 0.2
corr_on_plot = True

outfile = '{0}/Corr_H17L19_techreps_mutdiffsel'.format(dirs['plots'])
plots.append(outfile)
PlotCorrelations(L1_tech_reps[0], L1_tech_reps[1], outfile, 'Library 1', 'Library 1 replicate', markersize = markersize, alpha = alpha, corr_on_plot=corr_on_plot)

outfile = '{0}/Corr_H17L19_biorep12_mutdiffsel'.format(dirs['plots'])
plots.append(outfile)
PlotCorrelations(bio_reps[0], bio_reps[1], outfile, 'Library 1', 'Library 2', markersize = markersize, alpha = alpha, corr_on_plot=corr_on_plot)

outfile = '{0}/Corr_H17L19_biorep13_mutdiffsel'.format(dirs['plots'])
plots.append(outfile)
PlotCorrelations(bio_reps[0], bio_reps[2], outfile, 'Library 1', 'Library 3', markersize = markersize, alpha = alpha, corr_on_plot=corr_on_plot)

outfile = '{0}/Corr_H17L19_biorep23_mutdiffsel'.format(dirs['plots'])
plots.append(outfile)
PlotCorrelations(bio_reps[1], bio_reps[2], outfile, 'Library 2', 'Library 3', markersize = markersize, alpha = alpha, corr_on_plot=corr_on_plot)

ShowPDF([f + '.pdf' for f in plots])
```

Correlations between replicates for H17-L10:

In [10]:

```
L10_reps = ['{0}/L1_H17L10_vs_L1_mock_r1_sitediffsel.txt'.format(dirs['diffsel']),
           '{0}/L2_H17L10_vs_L2_mock_sitediffsel.txt'.format(dirs['diffsel']),
           '{0}/L3_H17L10_vs_L3_mock_sitediffsel.txt'.format(dirs['diffsel'])]

plots = []

outfile = '{0}/Corr_H17L10_biorep12'.format(dirs['plots'])
plots.append(outfile)
PlotCorrelations(L10_reps[0], L10_reps[1], outfile, 'Library 1', 'Library 2')

outfile = '{0}/Corr_H17L10_biorep13'.format(dirs['plots'])
plots.append(outfile)
PlotCorrelations(L10_reps[0], L10_reps[2], outfile, 'Library 1', 'Library 3')

outfile = '{0}/Corr_H17L10_biorep23'.format(dirs['plots'])
plots.append(outfile)
PlotCorrelations(L10_reps[1], L10_reps[2], outfile, 'Library 2', 'Library 3')

ShowPDF([f + '.pdf' for f in plots])
```

Correlations between replicates for H17-L7:

In [11]:

```
L7_reps = ['{0}/L1_H17L7_vs_L1_mock_r1_sitediffsel.txt'.format(dirs['diffsel']),
           '{0}/L2_H17L7_vs_L2_mock_sitediffsel.txt'.format(dirs['diffsel']),
           '{0}/L3_H17L7_vs_L3_mock_sitediffsel.txt'.format(dirs['diffsel'])]

plots = []

outfile = '{0}/Corr_H17L7_biorep12'.format(dirs['plots'])
plots.append(outfile)
PlotCorrelations(L7_reps[0], L7_reps[1], outfile, 'Library 1', 'Library 2')

outfile = '{0}/Corr_H17L7_biorep13'.format(dirs['plots'])
plots.append(outfile)
PlotCorrelations(L7_reps[0], L7_reps[2], outfile, 'Library 1', 'Library 3')

outfile = '{0}/Corr_H17L7_biorep23'.format(dirs['plots'])
plots.append(outfile)
PlotCorrelations(L7_reps[1], L7_reps[2], outfile, 'Library 2', 'Library 3')

ShowPDF([f + '.pdf' for f in plots])
```

Correlations between replicates for H18-S415

In [12]:

```
H18S415_reps = ['{0}/L1_H18S415_vs_L1_mock_r1_sitediffsel.txt'.format(dirs['diffsel']),
           '{0}/L2_H18S415_vs_L2_mock_sitediffsel.txt'.format(dirs['diffsel']),
           '{0}/L3_H18S415_vs_L3_mock_sitediffsel.txt'.format(dirs['diffsel'])]

plots = []

outfile = '{0}/Corr_H18S415_biorep12'.format(dirs['plots'])
plots.append(outfile)
PlotCorrelations(H18S415_reps[0], H18S415_reps[1], outfile, 'Library 1', 'Library 2')

outfile = '{0}/Corr_H18S415_biorep13'.format(dirs['plots'])
plots.append(outfile)
PlotCorrelations(H18S415_reps[0], H18S415_reps[2], outfile, 'Library 1', 'Library 3')

outfile = '{0}/Corr_H18S415_biorep23'.format(dirs['plots'])
plots.append(outfile)
PlotCorrelations(H18S415_reps[1], H18S415_reps[2], outfile, 'Library 2', 'Library 3')

ShowPDF([f + '.pdf' for f in plots])
```

## average differential selection across replicate experiments with independent mutant virus libraries¶

In [13]:

```
def AverageDiffsel(infiles, outmutdiffselfile, outsitediffselfile):
    subprocess.check_call(['dms_merge',
                           outmutdiffselfile,
                           'average'] + infiles + 
                          ['--sitediffselfile', outsitediffselfile
                          ])
```

In [14]:

```
# for H17-L19, average the technical replicates of library 1, then average across biological replicates. Do this for each concentration of H17-L19:
for conc in [1,2,3]:
    # average technical replicates of library 1 at each concentration
    files = ['{0}/L1_H17L19_c{1}_r1_vs_L1_mock_r1_mutdiffsel.txt'.format(dirs['diffsel'], conc),
             '{0}/L1_H17L19_c{1}_r2_vs_L1_mock_r2_mutdiffsel.txt'.format(dirs['diffsel'], conc)]
    L1_meanmutfile = '{0}/L1_avg_H17L19_c{1}_mutdiffsel.txt'.format(dirs['diffsel'], conc)
    L1_meansitefile = L1_meanmutfile.replace('mutdiffsel.txt','sitediffsel.txt')
    AverageDiffsel(files, L1_meanmutfile, L1_meansitefile)
    
    # average biological replicates, using the average of library 1 technical replicates + library 2 + library 3
    files = ['{0}/L2_H17L19_c{1}_vs_L2_mock_mutdiffsel.txt'.format(dirs['diffsel'], conc),
             '{0}/L3_H17L19_c{1}_vs_L3_mock_mutdiffsel.txt'.format(dirs['diffsel'], conc),
             L1_meanmutfile]

    avg_meanmutfile = '{0}/biorep_avg_H17L19_c{1}_mutdiffsel.txt'.format(dirs['diffsel'], conc)
    avg_meansitefile = avg_meanmutfile.replace('mutdiffsel.txt','sitediffsel.txt')
    AverageDiffsel(files, avg_meanmutfile, avg_meansitefile)
```

In [15]:

```
# for H17-L10, H17-L7 and H18-S415, average across the three biological replicates:
for mab in ['H17L10', 'H17L7', 'H18S415']:
    files = ['{0}/L{1}_{2}_vs_L{1}_mock_mutdiffsel.txt'.format(dirs['diffsel'], lib, mab) for lib in [2,3]] + \
            ['{0}/L1_{1}_vs_L1_mock_r1_mutdiffsel.txt'.format(dirs['diffsel'], mab)]
    meanmutfile = '{0}/biorep_avg_{1}_mutdiffsel.txt'.format(dirs['diffsel'], mab)
    meansitefile = meanmutfile.replace('mutdiffsel.txt','sitediffsel.txt')
    AverageDiffsel(files, meanmutfile, meansitefile)
```

## plot correlations between concentrations of H17-L19¶

In [16]:

```
concentration_diffsels = ['{0}/biorep_avg_H17L19_c1_sitediffsel.txt'.format(dirs['diffsel']),
                          '{0}/biorep_avg_H17L19_c2_sitediffsel.txt'.format(dirs['diffsel']),
                          '{0}/biorep_avg_H17L19_c3_sitediffsel.txt'.format(dirs['diffsel'])]
plots = []

markersize = 6
corr_on_plot=True

outfile = '{0}/Corr_H17L19_c1c2'.format(dirs['plots'])
plots.append(outfile)
PlotCorrelations(concentration_diffsels[0], concentration_diffsels[1], outfile, '0.5 ug/ml', '1.0 ug/ml', markersize = markersize, corr_on_plot=corr_on_plot)

outfile = '{0}/Corr_H17L19_c2c3'.format(dirs['plots'])
plots.append(outfile)
PlotCorrelations(concentration_diffsels[1], concentration_diffsels[2], outfile, '1.0 ug/ml', '10.0 ug/ml', markersize = markersize, corr_on_plot=corr_on_plot)

outfile = '{0}/Corr_H17L19_c1c3'.format(dirs['plots'])
plots.append(outfile)
PlotCorrelations(concentration_diffsels[0], concentration_diffsels[2], outfile, '0.5 ug/ml', '10.0 ug/ml', markersize = markersize, corr_on_plot=corr_on_plot)


ShowPDF([f + '.pdf' for f in plots])
```

## plot differential selection across all sites in HA¶

In [17]:

```
def PlotPosDiffselBySiteSubplots(sitediffsel_files, sites, numcols, numrows, 
                                outfile=False, ylims=False, xlims=False,
                                figsize_horizontal = 12, figsize_vertical = 8,
                                bar_width = 1, custom_xticks=None):
    '''
    Plot positive differential selection for a given set of sites for several samples arranged in subplots.
    provide *sitediffsel.txt to be plotted left-to-right, top-to-bottom.
    numcols * numrows = len(codon_counts_files)
    '''
    
    # check inputs
    assert len(sitediffsel_files) == numcols*numrows
    
    fig, axes = plt.subplots(nrows=numrows, ncols=numcols, 
                             sharex='col', sharey='row', figsize=(figsize_horizontal,figsize_vertical))
    
    for i,ax in enumerate(axes.flatten()):
                
        # get the data:
        diffsel = pd.read_csv(sitediffsel_files[i])

        # extract the positive diffsel values for each site in the list of *sites*:
        pos_diffsels = [ float(diffsel.loc[diffsel['site'] == r]['positive_diffsel'])  for r in sites]

        ax.bar(sites, pos_diffsels, bar_width, color = 'k', alpha = 1, linewidth=0.1, align='center')

        if ylims:
            ax.set_ylim(bottom=ylims[0], top=ylims[1])
        else:
            pass
        if xlims:
            ax.set_xlim(bottom=xlims[0], top=xlims[1])
        else:
            ax.set_xlim(left=min(sites), right=max(sites))
    
    # clean up axes
    spineOffset = {'left': 2, 'bottom': 2}
    for ax in axes.reshape(-1):
        [spine.set_position(('outward',spineOffset[loc])) if loc in ['left','bottom'] else spine.set_color('none') for loc, spine in ax.spines.items() ] 
        ax.tick_params(axis='x', direction='out')
        ax.tick_params(axis='y', direction='out')
        ax.xaxis.set(ticks_position = 'bottom',  major_locator = MaxNLocator(len(sites), prune=None))
        ax.yaxis.set(ticks_position = 'left', major_locator = MaxNLocator(4, prune=None))
        
    # remove tick labels for all:
    for ax in axes.reshape(-1):
        for xlabel_i in ax.get_xticklabels():
            xlabel_i.set_visible(False)
        for ylabel_i in ax.get_yticklabels():
            ylabel_i.set_visible(False)
    
    # add tick labels to left and bottom:
    if numcols == 1:
        for i, xlabel_i in enumerate(axes[-1].get_xticklabels()):
            xlabel_i.set_visible(True)
            plt.setp(xlabel_i, rotation=0, fontsize=11)
    else:
        for ax in axes[-1]:
            for i, xlabel_i in enumerate(ax.get_xticklabels()):
                xlabel_i.set_visible(True)
                plt.setp(xlabel_i, rotation=0, fontsize=11)

    if numcols == 1:
        for ax in axes:
            for ylabel_i in ax.get_yticklabels():
                ylabel_i.set_visible(True)
    else:
        for ax in axes[:,0]:
            for ylabel_i in ax.get_yticklabels():
                ylabel_i.set_visible(True)
                
    # override with custom xticks and labels if provided
    if custom_xticks is not None:
        plt.xticks(custom_xticks, custom_xticks)

    filepath = '{0}/test_diffselbarplots.pdf'.format(dirs['plots'])
    if outfile:
        filepath = outfile
    plt.savefig(filepath)
    plt.close()
```

plots for technical and biological replicates of H17-L19 at concentration 3; for figure 2.

In [18]:

```
outfile = '{0}/PosDiffselbysite_H17L19_c3_replicates.pdf'.format(dirs['plots'])
numcols = 1
numrows = 4

# left-to-right, top-to-bottom order for subplots
sitediffsel_files = ['{0}/L1_H17L19_c3_r1_vs_L1_mock_r1_sitediffsel.txt'.format(dirs['diffsel']),
                     '{0}/L1_H17L19_c3_r2_vs_L1_mock_r2_sitediffsel.txt'.format(dirs['diffsel']),
                     '{0}/L2_H17L19_c3_vs_L2_mock_sitediffsel.txt'.format(dirs['diffsel']),
                     '{0}/L3_H17L19_c3_vs_L3_mock_sitediffsel.txt'.format(dirs['diffsel']),]

sites = range(2, 565) #
custom_xticks = [100,200,300,400,500]

PlotPosDiffselBySiteSubplots(sitediffsel_files, sites, numcols, numrows, outfile=outfile, 
                            ylims=(0,120), figsize_horizontal = 4, figsize_vertical = 8,
                           bar_width=1, custom_xticks=custom_xticks)

ShowPDF(outfile, width=300)
```

plots for dose-response of H17-L19:

In [19]:

```
outfile = '{0}/PosDiffselbysite_H17L19doseresponse.pdf'.format(dirs['plots'])
numcols = 1
numrows = 4

# left-to-right, top-to-bottom order for subplots
sitediffsel_files = ['{0}/L1_mockr1_vs_mockr2_sitediffsel.txt'.format(dirs['diffsel']),
                     '{0}/biorep_avg_H17L19_c1_sitediffsel.txt'.format(dirs['diffsel']),
                     '{0}/biorep_avg_H17L19_c2_sitediffsel.txt'.format(dirs['diffsel']),
                     '{0}/biorep_avg_H17L19_c3_sitediffsel.txt'.format(dirs['diffsel']),]

sites = range(2, 565) #
custom_xticks = [100,200,300,400,500]

PlotPosDiffselBySiteSubplots(sitediffsel_files, sites, numcols, numrows, outfile=outfile, 
                            ylims=(0,120), figsize_horizontal = 4, figsize_vertical = 8,
                           bar_width=1, custom_xticks=custom_xticks)

ShowPDF(outfile, width=300)
```

plots for all mAbs:

In [20]:

```
# four mAbs:
outfile = '{0}/PosDiffselbysite_allmabsBiorepAvg.pdf'.format(dirs['plots'])
numcols = 1
numrows = 4

# left-to-right, top-to-bottom order for subplots
sitediffsel_files = ['{0}/biorep_avg_H17L19_c3_sitediffsel.txt'.format(dirs['diffsel']),
                     '{0}/biorep_avg_H17L10_sitediffsel.txt'.format(dirs['diffsel']),
                     '{0}/biorep_avg_H17L7_sitediffsel.txt'.format(dirs['diffsel']),
                     '{0}/biorep_avg_H18S415_sitediffsel.txt'.format(dirs['diffsel']),]

sites = range(2, 565) #
custom_xticks = [100,200,300,400,500]

PlotPosDiffselBySiteSubplots(sitediffsel_files, sites, numcols, numrows, outfile=outfile, 
                            figsize_horizontal = 4, figsize_vertical = 8,
                            bar_width=1, custom_xticks=custom_xticks)

ShowPDF(outfile, width=300)
```

## make logoplots of differential selection¶

In [21]:

```
def DiffselLogoplots(infiles, nperline='80', numberevery='3', restrictdiffsel='positive', 
                    colormap='jet', suffix=None):
    for infile in infiles:
        if suffix:
            outfile = infile.replace('.txt','{0}_logoplot.pdf'.format(suffix))
        else:
            outfile = infile.replace('.txt','_logoplot.pdf')
        subprocess.check_call(['dms_logoplot', infile, outfile,
                               '--nperline', nperline, '--numberevery', numberevery, 
                               '--restrictdiffsel', restrictdiffsel, '--colormap', colormap,
                               '--mapmetric', 'functionalgroup',
                               '--nosepline',
                               '--diffselheight'] + infiles)
```

In [22]:

```
# all four experimental replicates for H17-L19 at concentration 3 (two technical replicates of library 1, and library 2 and 3) equally scalled (for Figure 2):
replicate_diffsel_files = ['{0}/L1_H17L19_c3_r1_vs_L1_mock_r1_mutdiffsel.txt'.format(dirs['diffsel']),
                           '{0}/L1_H17L19_c3_r2_vs_L1_mock_r2_mutdiffsel.txt'.format(dirs['diffsel']),
                           '{0}/L2_H17L19_c3_vs_L2_mock_mutdiffsel.txt'.format(dirs['diffsel']),
                           '{0}/L3_H17L19_c3_vs_L3_mock_mutdiffsel.txt'.format(dirs['diffsel']),
                          ]
DiffselLogoplots(replicate_diffsel_files, suffix='four_replicates_scaled')
```

In [23]:

```
# mock_vs_mock and the three concentrations of H17-L19 (averaged), all equally scaled, for dose-response figure 3:
dose_response_diffsel_files = ['{0}/L1_mockr1_vs_mockr2_mutdiffsel.txt'.format(dirs['diffsel']),
                    '{0}/biorep_avg_H17L19_c1_mutdiffsel.txt'.format(dirs['diffsel']),
                    '{0}/biorep_avg_H17L19_c2_mutdiffsel.txt'.format(dirs['diffsel']),
                    '{0}/biorep_avg_H17L19_c3_mutdiffsel.txt'.format(dirs['diffsel'])]

DiffselLogoplots(dose_response_diffsel_files)
```

In [24]:

```
# biological replicate averages for all four mAbs, auto-scaling for each mab (Figure 4 main figure):
mab_diffsel_files = ['{0}/biorep_avg_H17L19_c3_mutdiffsel.txt'.format(dirs['diffsel']),
                     '{0}/biorep_avg_H17L7_mutdiffsel.txt'.format(dirs['diffsel']),
                     '{0}/biorep_avg_H17L10_mutdiffsel.txt'.format(dirs['diffsel']),
                     '{0}/biorep_avg_H18S415_mutdiffsel.txt'.format(dirs['diffsel']),
                     ]

for f in mab_diffsel_files:
    DiffselLogoplots([f], suffix='autoscale')
    
# individual replicates for each mAb besides H17L19, each trio equally scalled within each mAb:
for mab in ['H17L7', 'H17L10', 'H18S415']:
    mab_replicates = ['{0}/L1_{1}_vs_L1_mock_r1_mutdiffsel.txt'.format(dirs['diffsel'], mab),
                      '{0}/L2_{1}_vs_L2_mock_mutdiffsel.txt'.format(dirs['diffsel'], mab),
                      '{0}/L3_{1}_vs_L3_mock_mutdiffsel.txt'.format(dirs['diffsel'], mab),
                      ]
    DiffselLogoplots(mab_replicates, suffix='scaled_within_{0}_replicates'.format(mab))
```

## make pymol scripts to project max(mutdiffsel) to structure¶

In [25]:

```
def MapDiffselColorToSite(diffselfile, scriptfile, script_type='pymol', 
                          map_type='abs_diffsel', colors = ['#ffff99', '#990000'], sitecolorfile=None,
                          script_preamble=False, restrict_to_chain=False, prefsfile=None):
    '''Produces a colormapping based on differential selection and writes a script for `pymol` or `chimera`
    to color a structure by this colormapping.
    
    Uses the data in *diffselfile*, which can be either a mutdiffsel or a sitediffsel file, 
    depending on the specified *map_type*. 
    
    Writes a python script to *scriptfile* for the molecular visualization program specified 
    in *script_type* (either `pymol` or `chimera`).
    
    *colors* is a list of two colors defined by hex or r,g,b codes.
    
    How the mapping from differential selection to color is determined is specified by the 
    following choices for *map_type*:
    
        * `abs_diffsel`: plot total absolute differential selection at each site. 
        Total absolute differential selection will be converted to color by interpolating between 
        the two *colors*, which will be used to show the minimum and maximum values, respectively.
        Requries *diffselfile* to be a '*sitediffsel.txt' file.
        
        * `positive_diffsel`: plot the total positive differential selection at each site. 
        Absolute positive differential selection will be converted to color by interpolating between 
        the two *colors*, which will be used to show the minimum and maximum values, respectively.
        Requries *diffselfile* to be a '*sitediffsel.txt' file.
        
        * `negative_diffsel`: plot the total negative differential selection at each site. 
        Absolute negative differential selection will be converted to color by interpolating between 
        the two *colors*, which will be used to show the (absolute) minimum and maximum values, respectively.
        Requries *diffselfile* to be a '*sitediffsel.txt' file.
        
        * `max_pos_mutdiffsel`: plot the maximum positive mutdiffsel at each site (the value for the most 
        strongly enriched mutation at each site).
        Requries *diffselfile* to be a '*mutdiffsel.txt' file.
        
        * `positive_diffsel_by_Neff`: plot the total positive differential selection at each site,
        divided by Neff, the effective number of amino-acid mutations (exp(h) where h is the Shannon entropy of
        the amino-acid preference distribution at the site). This normalizes the total amount of
        differential selection at a site by the mutational tolerance at that site. If using this option, a
        path to a valid preferences file *prefsfile* must be provided as an additional keyword argument.
        
    
    If you want to restrict the recoloring to a single chain, specify the name of the chain to color to 
    *restrict_to_chain*. This currently only works for pymol scripts, not chimera scripts.
        
    Optionally, provide a path for a *sitecolorfile* to which the (site, hexcolor, rgbcolor) mappings are
    written.
    
    Optionally, if a *script_preamble* is provided, it is written to the top of the script before the 
    commands for colormapping. This may be a useful place to add other pymol or chimera commands to load 
    the pdb file, orient the view, etc.
    '''

    # read in the data from the diffselfile:
    df = pd.read_csv(diffselfile)
    df = df.dropna()
    column_names = list(df)
    if column_names == ['site', 'wt', 'mut', 'diffsel']:
        filetype = 'mutdiffsel'
    elif column_names == ['site', 'abs_diffsel', 'positive_diffsel', 'negative_diffsel']:
        filetype = 'sitediffsel'
    else:
        raise ValueError('diffsel file does not have appropriate set of column identifiers')
    
    # establish the color spectrum in hex and rgb.
    n_subdivisions = 500 # the color spectrum will be divided into this many discrete colors
    color1 = Color(colors[0])
    color2 = Color(colors[1])
    hex_spectrum = [c.hex for c in color1.range_to(color2, n_subdivisions)]
    hex_spectrum_dict = dict([(i, hex_spectrum[i]) for i in range(len(hex_spectrum))]) 
    rgb_spectrum = [c.rgb for c in color1.range_to(color2, n_subdivisions)]
    rgb_spectrum_dict = dict([(i, rgb_spectrum[i]) for i in range(len(rgb_spectrum))])
    
    # generate the site ==> colorindex mapping based on *map_type*:
    if map_type == 'abs_diffsel':
        assert filetype == 'sitediffsel'
        min_diff = df.min()['abs_diffsel']  
        max_diff = df.max()['abs_diffsel']  # the min and max will be mapped to color1 and color2, respectively
        range_diff = max_diff - min_diff
        df['colorindex'] =  (df.abs_diffsel - min_diff)/range_diff*(n_subdivisions-1)
        
    elif map_type == 'negative_diffsel':
        assert filetype == 'sitediffsel'
        abs_neg_col = abs(df['negative_diffsel'])
        df = df.assign(abs_neg=abs_neg_col)
        min_diff = df.min()['abs_neg']  
        max_diff = df.max()['abs_neg']  # the min and max will be mapped to color1 and color2, respectively
        range_diff = max_diff - min_diff
        df['colorindex'] =  (df.abs_neg - min_diff)/range_diff*(n_subdivisions-1)
            
    elif map_type == 'positive_diffsel':
        assert filetype == 'sitediffsel'
        min_diff = df.min()['positive_diffsel']  
        max_diff = df.max()['positive_diffsel']  # the min and max will be mapped to color1 and color2, respectively
        range_diff = max_diff - min_diff
        df['colorindex'] =  (df.positive_diffsel - min_diff)/range_diff*(n_subdivisions-1)
    
    elif map_type == 'positive_diffsel_by_Neff':
        assert filetype == 'sitediffsel'
        # get entropies, make Neff dict, and normalize positive_diffsel values by Neff:
        (sites, wts, pi_means, pi_95credint, h) = dms_tools.file_io.ReadPreferences(prefsfile)
        neff_dict = {} # keyed by int(site) so that it can be mapped to the diffsel dataframe. 
        # this will be problematic with non-integer site strings... 
        # maybe best fix will be to cast the site column of the dataframe as string,
        # and see if the map function will work with a dictionary of strings as sites.
        for site, entropy in h.iteritems():
            neff_dict[int(site)] = 2.**entropy
        df['neff'] = df['site'].map(neff_dict)
        df['positive_diffsel_by_Neff'] = df['positive_diffsel']/df['neff']
        
        min_diff = df.min()['positive_diffsel_by_Neff']  
        max_diff = df.max()['positive_diffsel_by_Neff']  # the min and max will be mapped to color1 and color2, respectively
        range_diff = max_diff - min_diff
        df['colorindex'] =  (df.positive_diffsel_by_Neff - min_diff)/range_diff*(n_subdivisions-1)
        
        # since there are no preferences for site 1, the colorindex is currently NaN when using Neff.
        # fill to 0.
        df['colorindex'] = df['colorindex'].fillna(0)
    
    elif map_type == 'max_pos_mutdiffsel':
        assert filetype == 'mutdiffsel'
        # sort mutdiffsel file by diffsel in case it wasn't already:
        df.sort_values('diffsel', ascending=False, inplace=True)
        # a new dataframe to only store the max mutdiffsel from each site
        newdf = pd.DataFrame(columns=['site', 'wt', 'mut', 'diffsel']) 
        for row in df.itertuples():
            if not any(newdf['site'].astype(int) == int(row[1])):
                newdf = newdf.append(pd.DataFrame([row[1:]], columns = ['site', 'wt', 'mut', 'diffsel']))
        newdf['site'] = newdf['site'].apply(int)
        # replace negative values with zero at sites where there was no positive selection:
        newdf = newdf.clip(lower=0)
        min_diff = newdf.min()['diffsel']  
        max_diff = newdf.max()['diffsel']  # the min and max will be mapped to color1 and color2, respectively
        range_diff = max_diff - min_diff
        newdf['colorindex'] =  (newdf.diffsel - min_diff)/range_diff*(n_subdivisions-1)
        df = newdf        

    else:
        raise ValueError("%s is not a recognized map_type." % map_type)
        
    # add a column for colors for each site    
    df['colorindex'] = df['colorindex'].astype(int) # round to nearest index
    df['hex'] = df['colorindex'].map(hex_spectrum_dict)
    df['rgb'] = df['colorindex'].map(rgb_spectrum_dict)        
    site_color_mapping = pd.concat([df['site'], df['hex'], df['rgb']], axis=1)
    
    if sitecolorfile:
        site_color_mapping.to_csv(sitecolorfile, index=False)
    
    # write out the script to *scriptfile*:
    f = open(scriptfile, 'w')
    
    if script_preamble:
        for line in script_preamble:
            f.write(line)
    
    if script_type == 'chimera':
        f.write("from chimera import runCommand\n")
        for i in range(len(df.index)):
            f.write("runCommand(color %s :%s)" % (site_color_mapping.iloc[i]['hex'], site_color_mapping.iloc[i]['site']) )
    elif script_type == 'pymol':
        for i in range(len(df.index)):
            rgblist = [min(1, c) for c in site_color_mapping.iloc[i]['rgb']]
            f.write("cmd.set_color(\'color%s\', \'%s\')\n" % (site_color_mapping.iloc[i]['site'], rgblist))
            if restrict_to_chain:
                f.write("cmd.color(\'color%s\', \'chain %s and resi %s\')\n" % (site_color_mapping.iloc[i]['site'], restrict_to_chain, site_color_mapping.iloc[i]['site']))
            else:
                f.write("cmd.color(\'color%s\', \'resi %s\')\n" % (site_color_mapping.iloc[i]['site'], site_color_mapping.iloc[i]['site']))
    else:
        raise ValueError("script_type must be chimera or pymol.")
    f.close()
```

In [26]:

```
colors = ('#fafafa', '#ff0000')

mutdiffselfiles = ['{0}/biorep_avg_H17L19_c3_mutdiffsel.txt'.format(dirs['diffsel']),
                   '{0}/biorep_avg_H17L10_mutdiffsel.txt'.format(dirs['diffsel']),
                   '{0}/biorep_avg_H17L7_mutdiffsel.txt'.format(dirs['diffsel']),
                   '{0}/biorep_avg_H18S415_mutdiffsel.txt'.format(dirs['diffsel']),
                  ]
mutmap_types = ['max_pos_mutdiffsel']

base_script_preample = [
    "cmd.set('bg_rgb','[1,1,1]')\n",
    "cmd.set('specular', '0')\n",
    "cmd.set('light_count','0')\n",
    "cmd.set('antialias','2')\n"]

view = (\
    -0.760245264,   -0.039225433,    0.648637414,\
    -0.649792910,    0.052100927,   -0.758461475,\
    -0.004043628,   -0.997895658,   -0.065079078,\
     0.001003772,   -0.004310774, -404.710021973,\
    77.289749146,   -5.298280716,   19.263229370,\
   319.299011230,  490.685546875,  -20.000000000 )

set_view = "cmd.set_view('%s')\n" % ', '.join([str(v) for v in view])
base_script_preample.append(set_view)

for f in mutdiffselfiles:
    for map_type in mutmap_types:
        outfile = f.replace('.txt','_%s_colormapping.py' % map_type)
        MapDiffselColorToSite(f, outfile, map_type = map_type, colors=colors, script_preamble=base_script_preample)
```

In [27]:

```
# for making figure zooming in on each epitope:

L19_zoom_view = (\
    -0.976966500,   -0.048147742,    0.208509833,\
    -0.212888569,    0.120258600,   -0.969767988,\
     0.021608414,   -0.991602182,   -0.127704829,\
    -0.014455989,   -0.001244724, -211.759735107,\
    60.558479309,   -2.605110168,  -15.578004837,\
   127.427650452,  298.814392090,  -20.000000000 )

L10_zoom_view = (\
    -0.829147160,   -0.013435517,    0.559087157,\
    -0.558676898,   -0.024620410,   -0.829147637,\
     0.024898469,   -0.999630868,    0.012910990,\
    -0.014740715,    0.000468306, -160.154312134,\
    77.069519043,   -3.746402740,  -21.436498642,\
    75.833206177,  247.219696045,  -20.000000000 )

Cb_zoom_view = (\
    -0.104112580,   -0.146713927,    0.983813584,\
    -0.993929207,   -0.022947490,   -0.108612485,\
     0.038506679,   -0.988943219,   -0.143396497,\
    -0.001266338,   -0.003537253, -220.251998901,\
    77.218444824,   -3.926383495,   -7.785912991,\
   136.186401367,  307.573211670,  -20.000000000 )
```
