# Supplementary material for: Complete mapping of viral escape from neutralizing antibodies
Source: PLoS Pathog. 2017 Mar 13;13(3):e1006271. doi: 10.1371/journal.ppat.1006271 (PMC5363992; doi:10.1371/journal.ppat.1006271)
Supplement: S1 Table — Percent infectivity was measured by qRT-PCR of the influenza nucleoprotein gene and interpolated from a standard curve of infection prepared with serial dilutions of each virus library. (PDF) [file ppat.1006271.s010.pdf]

	library 1	library 1 replicate	library 2	library 3
H17-L19 0.5 μg/ml	2.1%	2.5%	4.9%	5.4%
H17-L19 1 μg/ml	0.7%	0.7%	1.9%	1.8%
H17-L19 10 μg/ml	0.3%	0.2%	0.4%	0.4%
H17-L10 3 μg/ml	0.2%		0.2%	0.2%
H17-L7 15 μg/ml	0.2%		0.1%	0.1%
H18-S415 3 μg/ml	0.2%		0.1%	0.2%

S1 Table: Percentage of each mutant virus library remaining infectious after antibody neutralization in each replicate selection experiment. Percent infectivity was measured by qRT-PCR of the influenza nucleoprotein gene and interpolated from a standard curve of infection prepared with serial dilutions of each virus library.
