# Supplementary material for: Complete mapping of viral escape from neutralizing antibodies
Source: PLoS Pathog. 2017 Mar 13;13(3):e1006271. doi: 10.1371/journal.ppat.1006271 (PMC5363992; doi:10.1371/journal.ppat.1006271)
Supplement: S2 Table — Note that the classic experiments used the A/Puerto Rico/8/1934 (H1N1) virus, whereas our study used the A/WSN/1933 (H1N1) virus. In the older papers, multiple names were used to refer to the same antibody: H17-L19 was also called Ca3; H17-L10 was also called Ca6; H17-L7 was also called Cb15; H18-S415 was also called Cb5. (PDF) [file ppat.1006271.s011.pdf]

H17-L19

antigenic site	mutant virus name	mutation	WSN HA numbering
Ca2	DV4	S-P	153
Ca2	NV2	G-R	156
Ca2	NV7	S-G	158

H17-L10

antigenic site	mutant virus name	mutation	WSN HA numbering
Ca1	SV3	G-R	253
Ca1	WV8	S-L	220
Ca1	WV10	V-A	182
Ca1	WV11	G-R	186
Ca1	WV15	G-E	253
Ca1	ZV1	G-V	186

H17-L7

antigenic site	mutant virus name	mutation	WSN HA numbering
Cb	AV1	R-G	91
Cb	LV1	R-G	91
Cb	LV7	S-P	92
Cb	RV7	L-P	87

H18-S415

antigenic site	mutant virus name	mutation	WSN HA numbering
Cb	LV7	S-P	92
Cb	RV6	E-K	132
Cb	RV7	L-P	87

S2 Table: All mutations identified in the classic escape mutant selections with the four antibodies used in our study. Note that the classic experiments used the A/Puerto Rico/8/1934 (H1N1) virus, whereas our study used the A/WSN/1933 (H1N1) virus. In the older papers, multiple names were used to refer to the same antibody: H17-L19 was also called Ca3; H17-L10 was also called Ca6; H17-L7 was also called Cb15; H18-S415 was also called Cb5.
