# Supplementary material for: Complete mapping of viral escape from neutralizing antibodies
Source: PLoS Pathog. 2017 Mar 13;13(3):e1006271. doi: 10.1371/journal.ppat.1006271 (PMC5363992; doi:10.1371/journal.ppat.1006271)
Supplement: S3 Table — Sample names designate the mutant virus library used (L1, L2, or L3), the antibody used for selection (or mock in absence of antibody), the relative antibody concentration used if applicable (c1 is lowest, c3 is highest), and technical replicate (r1 or r2) if applicable. Targeted subamplicon barcode complexity refers to the number of uniquely barcoded molecules used in round 2 PCR (see methods) for each of the six HA subamplicons. Targeted total barcode complexity accounts for all six HA subamplicons. Total reads is the total number of paired-end sequencing reads obtained. Total aligned barcodes is the total number of barcodes (across all six subamplicons) that could be aligned with at least two paired-end sequencing reads. Median effective depth is the median number of barcodes aligned per HA codon. Previous deep sequencing of the input libraries used here [32] found at least three occurrences of between 47% and 51% of the total possible amino-acid mutations (over 97% of the possible amino-acid mutations were found at least three times in the starting plasmid mutant libraries before functional selection removed mutations incompatible with viral growth). (PDF) [file ppat.1006271.s012.pdf]

sample	targeted subamplicon barcode complexity	targeted total barcode complexity	total reads	total aligned barcodes	median effective depth
L1_H17L10	150000	900000	4041487	407110	66459
L1_H17L19_c1_r1	150000	900000	8695632	710204	114457
L1_H17L19_c1_r2	150000	900000	8624087	711932	111935
L1_H17L19_c2_r1	150000	900000	8741879	694883	108903
L1_H17L19_c2_r2	150000	900000	8511629	687655	108645
L1_H17L19_c3_r1	150000	900000	8192158	645638	103958
L1_H17L19_c3_r2	150000	900000	8442463	644335	101350
L1_H17L7	150000	900000	2897563	668184	110674
L1_H18S415	150000	900000	3639853	629643	101859
L1_mock_r1	700000	4200000	7533263	1601770	234020
L1_mock_r2	700000	4200000	8048792	1717683	261924
L2_H17L10	150000	900000	3932784	422093	69109
L2_H17L19_c1	150000	900000	5204031	498120	75830
L2_H17L19_c2	150000	900000	5515902	427803	66994
L2_H17L19_c3	150000	900000	5097722	415332	64041
L2_H17L7	150000	900000	3264651	664082	109002
L2_H18S415	150000	900000	3559630	711546	111575
L2_mock	500000	3000000	8802646	1341443	227037
L3_H17L10	150000	900000	2972786	657891	109015
L3_H17L19_c1	150000	900000	5073955	399545	64014
L3_H17L19_c2	150000	900000	5356951	402038	68581
L3_H17L19_c3	150000	900000	4080539	426897	68626
L3_H17L7	150000	900000	3704509	723491	118145
L3_H18S415	150000	900000	3376534	660742	106292
L3_mock	500000	3000000	9195791	1349386	226479

S3 Table: Summary statistics for barcoded subamplicon sequencing libraries. Sample names designate the mutant virus library used (L1, L2, or L3), the antibody used for selection (or mock in absence of antibody), the relative antibody concentration used if applicable (c1 is lowest, c3 is highest), and technical replicate (r1 or r2) if applicable. Targeted subamplicon barcode complexity refers to the number of uniquely barcoded molecules used in round 2 PCR (see methods) for each of the six HA subamplicons. Targeted total barcode complexity accounts for all six HA subamplicons. Total reads is the total number of paired-end sequencing reads obtained. Total aligned barcodes is the total number of barcodes (across all six subamplicons) that could be aligned with at least two paired-end sequencing reads. Median effective depth is the median number of barcodes aligned per HA codon. Previous deep sequencing of the input libraries used (Doud and Bloom, *Viruses*, 2016) here found at least three occurrences of between 47% and 51% of the total possible amino-acid mutations (over 97% of the possible amino-acid mutations were found at least three times in the starting plasmid mutant libraries before functional selection removed mutations incompatible with viral growth).
